# Scalable, Patternable Glass‐Infiltrated Ceramic Radiative Coolers for Energy‐Saving Architectural Applications

**DOI:** 10.1002/advs.202302701

**Published:** 2023-07-23

**Authors:** Seung Kyu Jeon, June Tae Kim, Min Seong Kim, In Soo Kim, Sung Jin Park, Hyeondeok Jeong, Gil Ju Lee, Yeong Jae Kim

**Affiliations:** ^1^ Ceramic Total Solution Center Korea Institute of Ceramic Engineering and Technology 3321, Gyeongchung‐daero, Sindun‐myeon, Icheon‐si, Gyeonggi‐do Icheon 17303 Republic of Korea; ^2^ Department of Electronics Engineering Pusan National University 2, Busandaehak‐ro 63 beon‐gil Busan 46241 Republic of Korea; ^3^ Nanophotonics Research Center Korea Institute of Science and Technology 5. Hwarang‐ro 14‐gil, Seongbuk‐gu Seoul 02792 Republic of Korea; ^4^ KIST‐SKKU Carbon‐Neutral Research Center Sungkyunkwan University (SKKU) Suwon 16419 Republic of Korea; ^5^ School of Advanced Materials Science and Engineering Sungkyunkwan University (SKKU) Suwon 16419 Republic of Korea; ^6^ Carbon Composite Materials Research Center Korea Institute of Science and Technology 92 Chudong‐ro, Bongdong‐eup Wanju‐gun Jeonbuk 55324 Republic of Korea

**Keywords:** glass infiltration, multi‐layer, non‐shrinkable ceramics, passive radiative cooling, thermal management

## Abstract

A huge concern on global climate/energy crises has triggered intense development of radiative coolers (RCs), which are promising green‐cooling technologies. The continuous efforts on RCs have fast‐tracked notable energy‐savings by minimizing solar absorption and maximizing thermal emission. Recently, in addition to spectral optimization, ceramic‐based thermally insulative RCs are reported to improve thermoregulation by suppressing heat gain from the surroundings. However, a high temperature co‐firing process of ceramic‐based thick film inevitably results in a large mismatch of structural parameters between designed and fabricated components, thereby breaking spectral optimization. Here, this article proposes a scalable, non‐shrinkable, patternable, and thermally insulative ceramic RC (SNPT‐RC) using a roll‐to‐roll process, which can fill a vital niche in the field of radiative cooling. A stand‐alone SNPT‐RC exhibits excellent thermal insulation (≈0.251 W m^−1^ K^−1^) with flame‐resistivity and high solar reflectance/long‐wave emissivity (≈96% and 92%, respectively). Alternate stacks of intermediate porous alumina/borosilicate (Al_2_O_3_‐BS) layers not only result in outstanding thermal and spectral characteristics, causing excellent sub‐ambient cooling (i.e., 7.05 °C cooling), but also non‐shrinkable feature. Moreover, a perforated SNPT‐RC demonstrates its versatility as a breathable radiative cooling shade and as a semi‐transparent window, making it a highly promising technology for practical deployment in energy‐saving architecture.

## Graphical abstract

The SNPT‐RC can be a promising solution for the scalability and durability issues of conventional RCs. By infusing borosilicate particles into a porous alumina layer, the structure shrinks much less in fabrication, is thermally insulative, and is physically durable while improving optical attributes efficient for radiative cooling. Our SNPT‐RC can have versatile usage, including fenestration or outer walls of buildings.

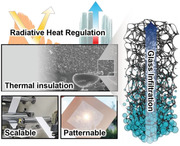

## 1 Introduction

Owing to global energy and climate crises, the idea of achieving carbon neutrality by 2050 is becoming a global consensus. This public opinion has spurred the development of green technology in wide‐ranging industries. Among them, buildings, which are essential, account for ≈40% of global energy consumption.^[^ ^1^ ^]^ From this perspective, eco‐friendly building materials for commercial and residential construction have been substantially developed to meet the global consensus of achieving net‐zero emissions. Recently, passive radiative cooling has gained attention as an attractive solution to address this challenging issue due to its advantages such as low cost,^[^ ^2^, ^3^ ^]^ compactness,^[^ ^4^, ^5^ ^]^ energy efficiency,^[^ ^6^, ^7^ ^]^ and zero carbon emissions.^[^ ^8^ ^]^ Passive radiative cooling takes advantage of strong reflectance in the solar spectrum region (0.3–2.5 µm) and high thermal emission in the long‐wave infrared region (LWIR; 8–13 µm). Radiative coolers (RCs) absorb heat from the object and radiatively emit it to the cold outer space (≈3 K) through an LWIR window. The importance of the spectral feature in RCs and the need for mass production have promoted plenty of research related to spectral optimization of RCs,^[^ ^9^, ^10^, ^11^, ^12^ ^]^ material engineering,^[^ ^13^, ^14^, ^15^, ^16^ ^]^ affordability,^[^ ^2^, ^11^ ^]^ and large‐scale production.^[^ ^3^, ^17^, ^18^ ^]^

In addition to these features, several researchers have reported that incorporating strong thermal insulation into RCs enhances their thermoregulation characteristics by suppressing heat gain from the surrounding environment.^[^ ^19^, ^20^ ^]^ Despite the usefulness of porous polymers in achieving spectral features and thermal insulation, their inherent mechanical and chemical instability can lead to degradation over time or under harsh conditions, such as exposure to hot weather or strong short‐wavelength irradiation. Moreover, their thermal stability is often inadequate for prolonged use in applications that demand high‐temperature resistance. Ceramic materials have emerged as strong candidates for radiative cooling materials due to their intrinsic flame retardability, corrosion resistance, and mechanical robustness.^[^ ^18^, ^21^, ^22^, ^23^, ^24^ ^]^ However, the high‐temperature annealing process required for ceramic materials unavoidably causes shrinkage of the fabricated sample, which not only reduces the overall sample size,^[^ ^25^ ^]^ but also compromises the optimal structure for spectral optimization (i.e., high solar reflection and thermal emission).^[^ ^26^ ^]^

Herein, we propose a scalable, non‐shrinkable, patternable, and thermally stable ceramic RC (SNPT‐RC) through scalable tape‐casting and co‐firing processes composed of alumina (Al_2_O_3_) and borosilicate (BS) glass composite. During the firing process, the BS glass undergoes melting, which then facilitates the shallow permeation of the molten glass into the Al_2_O_3_ matrix. This resulting glass infiltration enables the Al_2_O_3_ and BS glass layers to securely interlock with each other, thereby effectively preventing shrinkage. Also, abundant pores in multiple stacks of alumina/borosilicate (Al_2_O_3_‐BS) layers promote vertical thermal insulation and transverse heat dissipation simultaneously.^[^ ^27^ ^]^ In addition to high thermal insulation, these pores provoke multiple Mie scattering, increasing solar reflectivity remarkably. Based on process optimization and theoretical designs, an optimized SNPT‐RC is fabricated as the form of stand‐alone with excellent thermal insulation (≈0.251 W m^−1^ K^−1^) and high solar reflectance/long‐wave emissivity (≈96% and 92%, respectively). In outdoor measurements, the optimized SNPT‐RC shows exceptional sub‐ambient cooling capability (i.e., 7.05 °C cooling). Besides, the perforated and non‐shrinkage features of SNPT‐RC allow for the reliable production of breathable shades or windows with outstanding cooling performance, unlike other ceramic RCs that shrink during the co‐firing process.

## 2 Results and Discussion

A passive radiative cooling panel made entirely of ceramics is demonstrated in this study. The RC panel utilizes the spectral characteristic of an Al_2_O_3_ porous surface and the thermally insulative nature of a porous BS structure to control heat exchange. The porous form of SNPT‐RC functions as a high solar reflector and isolates thermally in the out‐of‐plane direction. At the same time, the SNPT‐RC radiates heat in the form of infrared waves to outer space through the atmospheric window. To allow for light transmission, a perforated surface can be formed with the advantage of a non‐shrinkage feature, making the panel suitable for exterior building material.

**Figure 1:**
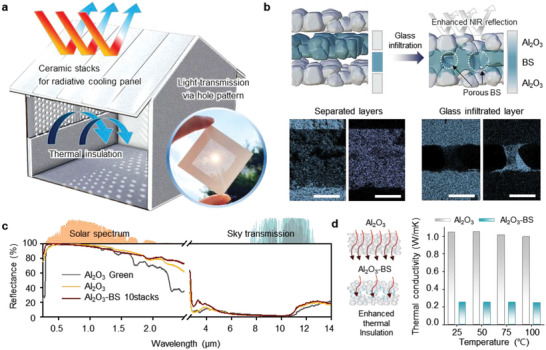
Ceramic stacks for a passive radiative cooling panel which featured light transmission and enhanced heat retention. a) 3D concept illustration and photographs of thermo‐insulative ceramic radiative cooling panel (SNPT‐RC) with perforated surface. b) The illustration and energy dispersive X‐ray analysis of a separated layer and glass infiltrated ceramic layer. c) The measured optical spectral reflectance at the solar spectrum and the sky transmission. d) The comparison of thermal conductivity between an alumina (Al_2_O_3_) monolayer and alumina/borosilicate (Al_2_O_3_‐BS) ceramic composites at 900 °C annealing temperature.

Controlling heat exchange is necessary for implementing passive cooling, and recent techniques pose maneuvers over this challenge by regulating radiative heat income and emission in a passive sense. The SNPT‐RC can play a critical role in a heat regulation, exploiting the spectral characteristic of an Al_2_O_3_ porous surface and the thermal insulative nature of porous BS structure (**Figure** **1**a). Specifically, the porous form of SNPT‐RC bi‐functionally works as a high solar reflector and thermal isolator in the out‐of‐plane direction. Simultaneously, the SNPT‐RC radiates a heat in the form of infrared (IR) waves to the outer space through atmospheric window. This work demonstrates a passive radiative cooling panel made entirely of ceramics. By utilizing the non‐shrinkage phenomenon, a perforated surface can be formed, allowing for the creation of a building exterior material that is capable of light transmission. Although previously researched RC structures, in which mostly porous or ceramic materials are solely utilized, show decent optical characteristics,^[^ ^28^, ^29^, ^30^ ^]^ practical difficulties still remain unsolved. Engagement of these two materials in particle scale could compensate the conventional RCs’ drawbacks, which are namely physical deformation of structure both in macroscopic and microscopic^[^ ^31^ ^]^ context, as ramifications of fabrication process. The SNPT‐RC comprises a BS layer sandwiched between two Al_2_O_3_ layers, as illustrated in Figure 1b. There are several potential benefits associated with this sandwiched structural design, including improvements in optical, thermal, and physical aspects. Since the Al_2_O_3_ porous layer encloses the lower surface of the BS layer, it is possible to stack additional BS‐Al_2_O_3_ interfaces, thereby facilitating reinforced multiple Mie scattering. Moreover, the stacked structure enhances thermal insulation between the inner and outer areas divided by the SNPT‐RC structure. In short, the sandwiched structural form can simultaneously provide enhancements in optical and thermal performance (Figure S1, Supporting Information). Through sintering process, BS particles infiltrate within Al_2_O_3_ layers, then they physically support each other and prevent further physical distortion while maintaining desired optical and adiabatic features, as explained further below.

Ideally, the optimal case for a radiative cooling structure is to maximize reflection toward solar heat radiation in the spectral range of ≈0.3–2.5 µm and focus radiative heat emission at the LWIR region of ≈8–13 µm.^[^ ^13^ ^]^ Measured spectral reflectance of three different structures (i.e., Al_2_O_3_ green, Al_2_O_3_, and Al_2_O_3_‐BS) are presented in Figure 1c. The Al_2_O_3_ green sample is the compound of poly‐vinyl alcohol (PVA) polymer and Al_2_O_3_ ceramic particles. PVA attributes high absorption of Al_2_O_3_ green sheet at the wavelength of 12 µm. During the sintering process in fabricating porous Al_2_O_3_ or Al_2_O_3_‐BS, PVA polymers are thermally decomposed, and these samples suffer slight increase (≈4%) in reflectance at the wavelength of ≈12 µm. Nevertheless, the SNPT structure exhibits a noticeable improvement in spectral reflectance, especially at solar spectral region. During the binder burn‐out process at 600 °C and annealing process at 900 °C of the Al_2_O_3_ green sheet, thermal decomposition of polymers occurs, resulting in vacancies within the entire structure and the formation of a porous structure consisting of pure Al_2_O_3_ ceramic particles. The presence of polymers leads to absorption in the ultraviolet and near‐infrared regions, causing a reduction in reflectance in the wavelength range of ≈0.78–2.5 µm (Figure 1c, gray curve). The yellow curve represents the spectral reflectance of the annealed Al_2_O_3_ sheet, which is improved in the near‐infrared region compared to that of the Al_2_O_3_ green sheet. Despite the same sintering temperature, the Al_2_O_3_‐BS sheet facilitates the ingress of BS particles into the pores, in contrast to the Al_2_O_3_ monolith. The spectral reflectance of the Al_2_O_3_‐BS sample (Figure 1c, brown curve) shows a wider bandwidth of high reflectivity in the solar radiation region. This enhancement is ascribable to the pore size reduction achieved by melted BS particles partially filling the void spaces between Al_2_O_3_ particles in near interfaces.

To maximize the thermal radiation emitted into the universe, it is crucial for the surface to possess a significantly high LWIR emittance within the range of 8–13 µm. The detailed LWIR emittance analyses are shown in Figure S2, Supporting Information. Moreover, another crucial aspect of the optimal optical characteristic for a RC is the high emissivity, or low reflectance at long wave infrared (LWIR) realm, where the transmittance of the atmosphere is considerably high. By exploiting this spectral region, a RC can successfully convey radiative heat toward the outer space, which plays an important role as a heat sink. As the measured spectral reflectance implies, the samples we used (i.e., porous Al_2_O_3_ and Al_2_O_3_‐BS sample) exhibit significantly low reflectance at wavelength range of ≈8–13 µm, which facilitates the efficient radiative cooling effect. The simulation‐based and experimental optical analyses for Al_2_O_3_ ceramic are demonstrated in Figure S3 and Note S2, Supporting Information. By attaining both high reflectance at solar radiation realm and high emissivity at LWIR region, the SNPT‐RC sample can flourishingly evade heat absorption from sun and discharge heat toward cold space, in radiative manner.

Thermal insulation is also a significant factor in determining the performance of a radiative cooling structure. Due to the thermally insulative nature of BS ceramic material, the resulting SNPT‐RC structure exhibits better thermal insulation performance compared to the Al_2_O_3_ monolayer (as shown in Figure 1d). At various temperatures, the thermal conductivity of the porous Al_2_O_3_ layer is ≈1.0 W m^−1^ K^−1^, which is much higher than the nearly constant thermal conductivity of ≈0.251 W m^−1^ K^−1^ observed in the SNPT‐RC structure with the same annealing temperature. Despite slightly higher thermal conductivity compared to conventional polymers such as PVDF (≈0.185 W m^−1^ K^−1^), our structure incorporates a design that effectively suppresses thermal conduction by utilizing porous Al_2_O_3_ and BS glass materials, while also providing resistance against UV degradation and fire hazards. Figure S2b, Supporting Information demonstrates the thermal anisotropic properties of Al_2_O_3_‐BS, exhibiting different in‐plane and through plane thermal conductivities, unlike traditional monolithic structures such as polymer and Al_2_O_3_ monolayer. This thermal anisotropic characteristic offers significant advantages in terms of heat dissipation.^[^ ^32^, ^33^ ^]^ Additionally, we measured the thermal conductivity and actual temperature deviation of structures with two different amounts of BS stacks (i.e., 5 stacks and 10 stacks), as depicted in Figure S1e,f, Supporting Information. In the SNPT‐RC structure, BS glass sheets with a thickness of 80 microns or less have been observed to have a thermal conductivity of less than 0.3 W m^−1^ K^−1^. Besides, the practical spectral and thermal performance of the SNPT‐RC is examined under several conditions, imitating possible defilement cases that could occur in actual environmental circumstances. Figure S4, Supporting Information demonstrates the measured values of spectral reflectance and thermal conductivity with changing the configurations of contamination.

Based on the spectral and thermal features of the SNPT‐RC structure, the potential energy savings for buildings in major cities with 19 climate zones are analyzed when the SNPT‐RC is applied to conventional building. The SNPT‐RC can save up to ≈3.5 GJ of cooling energy compared to a building with a conventional exterior tile (Figure S5a, Supporting Information). The detailed simulation geometry for the building is described in Figure S5b, Supporting Information. The global annually saved energy is also exhibited in Figure S5c, Supporting Information.

**Figure 2:**
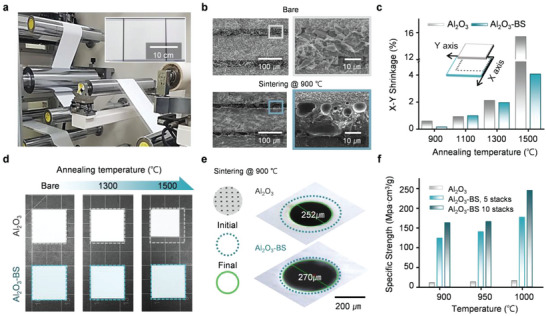
Mechanical features of ceramic composite after annealing process. a) Productive passive radiative ceramic composite fabricated by tape casting method for Al_2_O_3_ and BS materials. b) SEM images for Al_2_O_3_‐BS multilayer before/after annealing at 900 °C temperature. c) Plots of firing shrinkage rate for Al_2_O_3_ monolayer and Al_2_O_3_‐BS with varying annealing temperature. d) Photographs for shrinkage of Al_2_O_3_ monolith and Al_2_O_3_‐BS multilayered sheet. e) Microscope images of ceramic hole pattern for Al_2_O_3_ monolith and Al_2_O_3_‐BS multilayered sheet. f) Specific strength for Al_2_O_3_ monolith and Al_2_O_3_‐BS multilayered sheet.

Along with the advancement in optical attributes and thermal insulative features, structural combination of BS with porous Al_2_O_3_ structure can enhance mechanical characteristics of the SNPT‐RC. **Figure** **2**a presents a photograph of SNPT‐RC roll‐to‐roll process for mass production. A fabrication of the sample structure includes the tape casting method followed by the annealing process; first constructing each Al_2_O_3_ and BS layer, then stacking layers in Al_2_O_3_‐BS‐Al_2_O_3_ order, merging three layers through lamination and firing total ceramic layer in the end. Figure S6 and Note S1, Supporting Information provides detailed fabrication process of the SNPT‐RC structure. Along the co‐firing process, BS particles permeate into the vacancies between Al_2_O_3_ particles at layer boundaries, reinforcing the physical strength of the porous structure. Figure 2b shows cross‐sectional SEM images of bare and co‐fired structures. Before heat treatment, particles at each layer maintain its original form. In annealing process at 900 °C, the BS glass frits melt and seep into Al_2_O_3_ layers, agglomerating with adjacent particles and form larger effective particle size in result. Wetting angles are measured with heating microscope images at varying annealing temperatures, as shown in Figure S7, Supporting Information. This provides context for determining the appropriate material and annealing temperature, with a detailed explanation of the optical and physical characteristics of the materials used in the experiment. From this reaction, particles seize each other and forestall further structural and geographical distortion, while improving physical properties. Figure 2c presents comparison of measured X‐Y shrinkage ratio between Al_2_O_3_ monolayered structure and SNPT‐RC structure through different annealing temperatures. SNPT‐RC shows up to four times less diminution in size compared to Al_2_O_3_ monolayered structure, especially for higher annealing temperature. Figure 2d,e shows actual sample size changes in similar manner. Reducing the annealing temperature can enhance the light scattering efficiency of Al_2_O_3_ by increasing its porosity with weak agglomeration (as shown in Figure S8, Supporting Information), although this can lead to a significant decrease in its mechanical strength. However, Figure 2f demonstrates that SNPT‐RC structures with a higher proportion of BS show a notable improvement in specific strength with optical features. Briefly, the non‐shrinking and physically durable features of SNPT‐RC originates from particle‐wise entanglement, which could substantially ameliorate performances of other RCs which only employ single ceramics or polymer structures.

**Figure 3:**
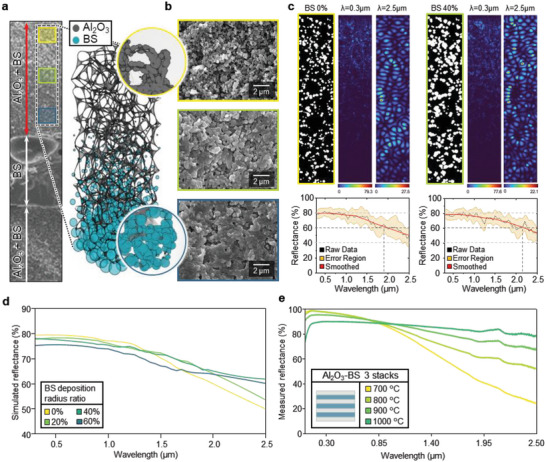
Optical simulation. a) Constructed approximated 3D model of Al_2_O_3_‐BS compound. The insets represent magnified view of two different region of the structure; the upper inset shows the upper area of porous structure composed of Al_2_O_3_ particles, and the lower inset shows BS particles deposited on the Al_2_O_3_ porous structure. b) Cross‐sectional SEM images of the SNPT structure with different proximity between Al_2_O_3_ and BS layer interface; top image (yellow) is farthest and bottom image (indigo) is closest to the interface. c) Electric‐field distributions and spectral responses for structures with BS 0% and 40% deposition. d) Simulated spectral reflectance of structures with different amount of BS deposition. e) Measured spectral reflectance of fabricated Al_2_O_3_‐BS sheets with different annealing temperature.

To investigate the optical influence of BS infiltration between Al_2_O_3_ nanoparticles, 2D finite‐difference time‐domain (FDTD) simulations are performed in terms of spectral reflectance, depending on the amount of the particle intrusion. The amount of BS deposition gradually diminishes as the position is further from the layer interface. **Figure** **3**a intuitively illustrates this nature of the SNPT structure in 3D model; where the Al_2_O_3_ base structure is constructed by utilizing Voronoi 3D method.^[^ ^34^ ^]^ As demonstrated in the model, porous Al_2_O_3_ base structure is conformed with Al_2_O_3_ nanoparticles connected in net‐like formation and the amount of BS deposition increases at the bottom area, at which the Al_2_O_3_ particles are close to BS layer interface.

Scanning electron microscopy (SEM) images of the cross‐sectional surface of the Al_2_O_3_ layer part of the SNPT structure are arranged in Figure 3b, which readily show the difference in BS agglomeration amount depending on the depth of its position. Detailed configurations related to FDTD simulation (i.e., boundary condition and grid size) are discussed in the experimental section. Figure S9a, Supporting Information displays the refractive indices of Al_2_O_3_ and BS, and Figure S9b, Supporting Information exhibits radius distribution of Al_2_O_3_ particles, and pores, whose counts are normalized by setting the maximum count values as one. For each deposition rate, 10 different random structures are generated while satisfying the statistical condition for particle/pore radii and counts, obtained in the SEM image analyzing process (Figures S10 and S11, Supporting Information). To properly implement the features of 3D structure on planar simulation domain, several statistics (i.e., number density and radius distribution of projected particles, outermost particles, and pores) are extracted from SEM cross‐sectional image and applied in 2D model generation, as specified in Figures S11 and S12, Supporting Information. With these models, spectral reflectance is simulated separately, and the average values with errors are displayed spectrally. The resultant average spectral reflectance is then smoothed for better visuality.

Simulation structures are discretely generated for each aggregation rate of BS, and electric field intensity simulation results are provided for two wavelength cases (Figure 3c); 0.3 and 2.5 µm wavelengths. The infiltration phenomenon of BS particles into the vacancies of Al_2_O_3_ nanoparticle layer is realized by overlaying BS particles on the Al_2_O_3_ particle bases. The simulation domain includes the structure with plane wave source and reflectance monitor (Figure S13c, Supporting Information). To consider the solar radiation spectrum, the wavelength of source is varied from 0.3 to 2.5 µm with step size of 0.05 µm. The electric field intensity profiles with different BS deposition rate (i.e., deposition radial width rate of 60%, 80%, and 100%, to the base Al_2_O_3_ particle radius) depicted in Figure S13d, Supporting Information shows the amount of wave propagated in the structures. Comparing the propagation distance of incident wave at 2.5 µm wavelength, the wave in the porous structure of pure Al_2_O_3_ propagates further into the deeper area, and total reflectance drops drastically. On the other hand, as the BS deposition radial width rate to Al_2_O_3_ particle radius increases to 100%, the distance of wave propagation shortens, and the resultant total reflection increases.

As the material and structural characteristics, the reflectance simulation results are presented in Figure 3d, which reveals the optical effect of BS infiltration. The BS percentage indicated in the legend of the graph represents the ratio of total BS deposition width to the base Al_2_O_3_ particle diameter, as depicted with sample particle models. For the pure porous Al_2_O_3_ structure case, reflectance remains high at short wavelength region ≈0.3 µm, but swiftly decays as the wavelength gets longer. As BS particles agglomerate on Al_2_O_3_ particles, even though the reflectance slightly drops at short wavelength regime, it tends to maintain its value compared to the former case, where the pores distributed through the entire simulation structure poises the overall spectral reflectance to yield high reflectance for wider wavelength bandwidth. Detailed simulation configurations and raw data of simulation results are demonstrated in Figure S13, Supporting Information.

Figure 3e shows measured spectral reflectance of the SNPT structure with varying annealing temperature. The structure annealed at 700 °C shows highest reflectance at short wavelength (i.e., ultra‐violet) regime, but it swiftly decays as the wavelength increases. At this temperature, BS cannot properly infiltrate into porous Al_2_O_3_ layer. On the other hand, for the structures which have undergone the annealing process with higher temperatures, reflectance subtly decreases at a wavelength range shorter than ≈1000 nm compared to the former but shows a higher tendency to maintain its value at a wavelength longer than ≈1000 nm. These improvements originate from the significant infiltration of BS.

Exhaustively considering these simulation results, the slight increase in spectral reflectance of the Al_2_O_3_‐BS structure at the infrared (IR) region, as shown in Figure 1c, can be attributed to the optical effect of BS infiltration. Specifically, as BS agglomerates on Al_2_O_3_ particles, the size of pores in the shallow region near the interface between the two layers decreases. At the IR wavelength range, the size of the pores becomes comparable to the wavelength, and they can be seen as particles with a refractive index similar to that of the surrounding air. This creates a high refractive index contrast between the Al_2_O_3_ particles, leading to enhanced Mie scattering phenomena at the boundary and a slight reinforcement of light reflectance across the entire structure.^[^ ^35^, ^36^ ^]^ However, since the BS infiltration occurs only at the adjoining region around the interface, the reflectance spectrum tends to resemble that of the porous Al_2_O_3_ structure. Thus, in Figure 1c, the spectral reflectance of the Al_2_O_3_‐BS structure is similar to that of pure Al_2_O_3_, but with a slight improvement at the IR region.

**Figure 4:**
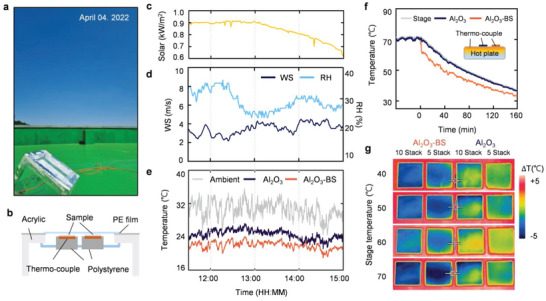
Daytime passive radiative cooling performance in outdoor experiments. a) Photograph of the measurement setup on the rooftop. b) 2D Schematic illustration of measurement set‐up. c) Real time recorded solar irradiance and d) wind speed (WS) and relative humidity (RH). e) Real time cooling performance for ambient air, Al_2_O_3_, and SNPT‐RC. f) Logged temperatures for hot plate stage, Al_2_O_3_, and SNPT‐RC. g) Thermographic images of samples depending on different stage temperatures.

Outdoor measurements of temperature over time were conducted at the rooftop of KICET (35°10′38.1“N 128°08′04.4”E), on daytime of April 4^th^, 2022. In order to confirm the cooling performance of the SNPT‐RC experimentally, the temperature of Al_2_O_3_ monolayered structure and SNPT‐RC structure were measured side by side (**Figure** **4**a). Experimental setup was composed of acrylic exterior and auxiliary measuring tools (Figure 4b). Polyethylene (PE) film covers upper area of the setup for blocking moisture being formed on sample and minimizing convective heat exchange, which could hinder accurate measurement on radiative cooling performance. Thermocouples are attached under samples, in order to quantify temperature differences from ambient temperature (thermocouple verification process is demonstrated in Figure S14, Supporting Information). An ambient air sensor was placed within an Al‐coated paper box to measure the temperature of the naturally convective air in the chamber. This setup effectively prevents overheating of the sensor due to solar irradiance. Additionally, a pyranometer (CMP6, Kipp & Zonen) was positioned adjacent to the acrylic chamber to measure solar irradiance. Last, polystyrene materials prevent thermal conduction between thermocouple and lower contacts. Figure 4c,d displays ambient data during measurement, such as solar irradiance, wind speed, and humidity. Figure 4e reveals decent cooling effect of designed SNPT‐RC structure. While the temperature of Al_2_O_3_ monolayered structure was measured to be ≈2–4 °C lower than ambient temperature, SNPT‐RC structure displays additional ≈2 °C cooling performance, especially under highest solar irradiance of ≈0.9 kW m^−2^. To examine the cooling performance of the sample under a practical scenario, detailed demonstration of complementary outdoor measurement without anti‐convection PE shield is presented in Figure S15, Supporting Information.

Additional experiment is conducted in the situations of hot lower contacts using hot plate to highlight thermal insulation feature of SNPT‐RC. The experiment is performed by measuring the temperatures of each sample (i.e., stage, Al_2_O_3_, and SNPT‐RC). Thermocouples are placed between hot plate (≈40–70 °C) and samples, and the hot plate is turned off at the temperature of 70 °C. Owing to superior thermal insulative feature of SNPT‐RC, its top surface temperature simultaneously drops as ≈1.5 °C compared to those of stage and Al_2_O_3_ structure when the hot plate is turned off (Figure 4f). The exceptional thermal insulation of SNPT‐RC hinders a heat transfer from the bottom to top surfaces, hence the temperature of SNPT‐RC top surface is relatively low. Figure 4g apparently shows the top surface temperature of each structure in accordance with listed stage temperature.

**Figure 5:**
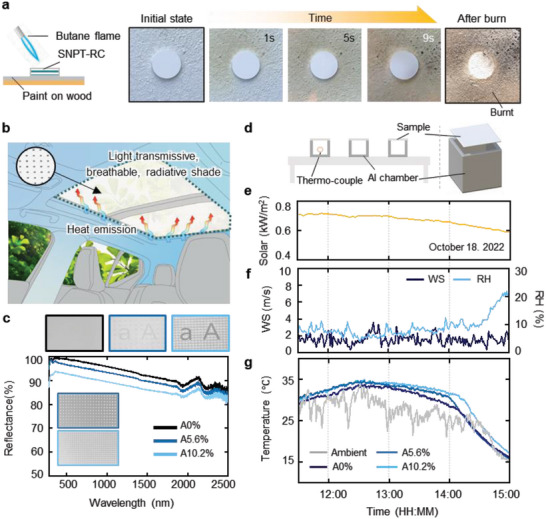
Radiative cooling performance of thermal insulative and perforated passive radiative cooling ceramic composite. a) Flame combustion experiments of the SNPT‐RC on paint/wood. b) Concept illustration of radiative cooling car sunshade application. c) The photographs and spectral reflectance results for different area ratio of hole patterns. d) Schematic illustration for outdoor measurement set‐ups with aluminum chamber. e–g) Out‐door measurement results between 11:30 and 15:00 from 18th October 2022. d) Schematic illustration for outdoor measurement set‐ups with aluminum chamber. e) Solar irradiance. f) Wind speed and humidity. g) The temperature of the samples measured outdoors.

Incorporating ceramic composites into the radiative cooling system/structure is beneficial for enhancing its thermal stability and making it more resistant to fire, as shown in **Figure** **5**a. A burning test was performed by applying a polymer‐based commercial paint onto wood and placing SNPT‐RC on top. The SNPT‐RC exhibited a much stronger ability to suppress flames, providing further support for its effectiveness in fire prevention. This advantage can help promote the engineering of radiative cooling in more practical circumstances. In the case of building materials, particularly for fire‐prone conditions, it is essential to ensure that their functionality is not compromised. However, polymer‐based materials undergo a color change when combusted, which can lead to a degradation in cooling performance due to the increased light absorption.^[^ ^37^, ^38^ ^]^ Conventional radiative cooling materials previously discussed up to this point have been lacking in fire‐resistive features, which could hinder the practical application of radiative cooling configurations in buildings.^[^ ^39^, ^40^, ^41^ ^]^ The SNPT‐RC endowed with improved combustion resistive‐ness can reinforce stability and robustness when implemented. Additionally, the structure can be applied to windows of stationary or mobile applications, in which solar radiation could heat the interior. As a potential application of SNPT‐RC, a structure with hole patterns could be used to allow better window visibility for automobiles (Figure 5b). Three representative cases were examined, with 0%, 5.6%, and 10.2% of the entire area opened via hole pattern, respectively, and their characteristics were investigated in terms of spectral reflectance and temperature over time. The through‐hole areal ratio calculation is demonstrated in Figure S16, Supporting Information. Figure 5c shows the reflectance spectra of the three samples in the solar spectrum. The inset in Figure 5c shows photographs of SNPT‐RC with different hole areas. To evaluate the cooling properties of a building space that utilizes a perforated surface cooling system, outdoor measurements were conducted with aluminum chamber, as illustrated in Figure 5d. The thermocouple was affixed to the bottom surface inside the chamber, while the emitter was positioned on the chamber's top surface. The ambient data were recorded simultaneously in daytime (Figure 5e,f). The logged temperature data indicates that the SNPT‐RC with a higher areal ratio of via holes exhibits poorer cooling performance (Figure 5g). This tendency is related to the lower spectral reflectance of samples with a higher areal hole ratio The 10.2% vacant structure (A10.2%) shows lower reflectance up to ≈7% and less cooling performance, about 1 °C higher than the filled structure. However, considering the importance of visibility for mobile windshields, the defect could be negligible, and applying a hole pattern can provide visibility of the window without significantly reducing cooling performance.

## 3 Conclusions

To make use of both optical features of Al_2_O_3_ porous structure and adiabatic characteristics of BS ceramic, we devised a SNPT‐RC by sequentially stacking Al_2_O_3_ and BS layers and sintering the whole structure, thus allowing permeation of BS particles into adjacent Al_2_O_3_ layers. As a passive heat regulator, SNPT‐RC can properly perform its role by vastly reflecting solar radiation and concentrating thermal emission at LWIR spectral range. From numerical analyses, infiltration of BS particles into gaps between particles enlarges effective particle diameter, thus inducing multiple scattering in a broader wavelength range at the solar spectrum, increasing reflection efficiency toward solar radiation. The fabricated SNPT‐RC showed sub‐ambient surface cooling performance of ≈4 °C, while Al_2_O_3_ monolayer structure only showed that of ≈2 °C.

In addition, the particle‐wise linkage of Al_2_O_3_ and BS advances the physical nature of the composition by successfully diminishing the shrinkage of the sample from the annealing process and improving the sample's specific strength. The SNPT structure showed up to four times less shrinkage and 15 times better specific strength compared to Al_2_O_3_ monolayered sample. Furthermore, we examined several samples with different areal coverages (i.e., areal percentage of hole patterns as 0%, 5.6%, and 10.2%) to check the applicability of SNPT structure at windshields of an automobile while properly performing enclosure cooling. We were able to conclude with the possibility of improving window visibility while maintaining cooling performance to an acceptable extent. Hence, the SNPT‐RC can be a potential solution for the pragmatic implementation of RCs, and its versatile application could raise cooling energy savings in various situations.

## 4 Experimental Section

### Calculation of Cooling Energy Saving

In the process of comparing annual cooling energy consumptions between conventional roof and the experimental structure, EnergyPlus (U.S. Department of Energy, version 22.2) was utilized as a whole‐building energy modeling engine. Overall simulation conditions and parameters were set according to following standards and references: American Society of Heating, Refrigerating and Air Conditioning Engineers (ASHRAE) standards and Typical Meteorological Year 3 (TMY3) climate data. Commercial medium sized office building from ASHRAE standard 90.1‐2019 was selected for physical structure under simulation. On top of that, material and roof profile were added and adjusted for implementing additional outmost SNPT‐RC layer on building roof. Solar and thermal absorptance of additional SNPT‐RC layer were configured as spectral average value (≈0.2–2.5 and ≈4–20 µm, respectively). Last, for climate conditions for simulation, weather data in ASHRAE standard were replaced with that of global representative locations. ASHRAE reference data include simulation profiles of 19 different climate zones (from 0 to 8 A), and TMY3 climate data of global representative locations (Table S1, Supporting Information) were used for each of those climate zones.

### Optical Simulations

A commercial software, FullWAVE (RSoft Design Group, Synposys, United States) revealed the total reflectance of Al_2_O_3_ nanoparticle layer. To streamline the calculation process, 2D simulation was chosen, and proper assumptions were made for the valid implementation of the 3D structure in 2D simulation by creating equivalent cross‐sectional planar model (Figures S10–S12, Supporting Information). Particularly, this work obtained particle and pore distribution from cross‐sectional SEM images, and generated 2D simulation model by arranging particles’ position and radii in which the resultant distribution fits the entire model into the pore and particle statistics.^[^ ^42^, ^43^, ^44^, ^45^ ^]^ The simulation domain was set as 10 × 50 µm^2^, and the grid size for FDTD simulation was 0.02 µm for each axis (i.e., x and z directions). Boundary conditions were set as periodic for x‐direction and perfectly matched layer (PML) for z‐direction.

In order to numerically derive the spectral reflectance of the SNPT structure with different amount of a particle deposition, BS particles were overlaid on Al_2_O_3_ particle bases, whose radial deposition width was defined in terms of its base particle diameter as follows:

(1) $$\mathrm{BS}\;\mathrm{deposition}\;\mathrm{radial}\;\mathrm{width}=\left(0.1\times p\right)\times \;\left({\mathrm{base}\;\mathrm{Al}}_{2}O_{3}\;\mathrm{particle}\;\mathrm{radius}\right)$$

The parameter *p* is set as a radial deposition ratio whose value increases from 0 to 10 with an interval of 2, which represents the gradual increasement of BS particle deposition on Al_2_O_3_ base particles for every direction. Each of the cases are simulated in wavelength region of ≈0.3–2.5 µm. For the precise numerical analysis, 10 random pore filters and corresponding planar simulation models were generated for each of *p* values, and resultant spectral reflectance were expressed as the average spectral values with error intervals and smoothed plot. (Figure S13, Supporting Information)

### Structural, Optical, and Thermal Analysis

The morphology of the fabricated cooling panel was observed using a scanning electron microscope (SEM, Hitachi S‐4800). Thermal and mechanical properties were characterized by measuring thermal diffusivity, specific heat, and thermal conductivity using a high‐temperature thermal conductivity measurement instrument (LFA467, Netzsch) in the temperature range of 25–100 °C. Additionally, the strength of the panel was evaluated using a modulus of rupture (MOR) tester, fabricating panels with dimensions of 3 × 4 × 35 mm^3^, where the upper and lower surfaces were parallel. The support span distance was set at 30 ± 0.1 mm, and the crosshead speed was set at 0.5 mm min^−1^. The UV–vis‐NIR and far IR spectrum was measured using a spectrophotometer (solidspec‐3700, Shimadzu Co.) and FT‐IR spectrometer (Nicolet iS50 FTIR, Thermo‐Fisher Scientific Co.) equipped with an integrating sphere.

## Conflict of Interest

The authors declare no conflict of interest.

## Supporting information

Supporting Information

## Data Availability

The data that support the findings of this study are available in the supplementary material of this article.
