# Supplementary material for: Scalable, Patternable Glass‐Infiltrated Ceramic Radiative Coolers for Energy‐Saving Architectural Applications
Source: Adv Sci (Weinh). 2023 Jul 23;10(27):2302701. doi: 10.1002/advs.202302701 (PMC10520670; doi:10.1002/advs.202302701)
Supplement: Supplementary file 1 — Supporting Information [file ADVS-10-2302701-s001.pdf]

ADVANCED SCIENCE Open Access

Supporting Information

for *Adv. Sci.*, DOI 10.1002/advs.202302701

Scalable, Patternable Glass-Infiltrated Ceramic Radiative Coolers for Energy-Saving Architectural Applications

Seung Kyu Jeon, June Tae Kim, Min Seong Kim, In Soo Kim, Sung Jin Park, Hyeondeok Jeong, Gil Ju Lee* and Yeong Jae Kim**

Supporting Information

Scalable, Patternable Glass-Infiltrated Ceramic Radiative Coolers for Energy-Saving Architectural Applications

Seung Kyu Jeon†, June Tae Kim†, Min Seong Kim, In Soo Kim, Sung Jin Park, Hyeondeok Jeong, Gil Ju Lee* and Yeong Jae Kim**

*Corresponding authors: Hyeondeok Jeong, Gil Ju Lee and Yeong Jae Kim

SI Note 1. Fabrication process for SNPT-RC

Figure S6 shows the detailed fabrication process of a ceramic passive cooling panel. The SNPT-RC was fabricated using a multilayer structure of alumina (Al_2O_3) and borosilicate glass (BS), employing a glass infiltration and tape casting method. The solvent mixture consisted of 60 vol.% toluene and 40 vol.% ethanol, and a dispersant, BYK-111 (BYK Chemical, Germany), was added at a concentration of 1 wt% to ensure slurry dispersion. The binder system used polyvinyl butyral (PVB, Sekisui, Japan) as a binder and dibutyl phthalate (DBP, Dejung, Korea) as a plasticizer, with a fixed plasticizer/binder ratio of 0.4. The binder content was prepared by adding 30 vol.% of Al_2O_3 (Sumitomo chemical Co., Japan) and 28 vol.% of borosilicate glass to the slurry. The raw materials for borosilicate glass (BS) are composed of 81.0 wt% SiO_2, 12.5 wt% of B_2O_3 4.0 wt% Na_2O and 2.5 wt% of Al_2O_3 and dry-mixed using a powder mixer at 700rpm for 15 minutes. Subsequently, the mixture underwent a melting process in a high-temperature lift-bottom furnace at 1500°C for 30 minutes. The resulting glass melt was then obtained through a water quenching method. The quenched glass frit underwent a size reduction process through dry grinding using a disk mill and wet grinding using a ball mill. It was further finely ground using an attrition mill to manufacture BS frit.

The manufactured BS frit was analyzed for particle size distribution using particle size analysis (PSA), microstructure using scanning electron microscope (SEM), and phase analysis using X-ray diffraction (XRD). As a result, an amorphous BS frit with a particle size of 1.6 μm was produced. The prepared ceramic powder, dispersant, and solvent were mixed using a 10 mm diameter zirconia ball in a ball mill for 24 hours for primary milling. Subsequently, additional solvent, binder, and plasticizer were added, and secondary milling was carried out for 24 hours. After removing the gas bubbles within the slurry through a defoaming process for 15 minutes, the slurry was stabilized through ball milling at a speed of 25 rpm for approximately 24 hours, resulting in a slurry viscosity of around 2000 cps. The Al_2O_3 and BS sheets were

shaped using a tape caster based on the doctor blade method of tape casting. The casting conditions included a casting speed of 2 m/min and a three-zone drying temperature profile of 35-60-75°C. The tape width was 150 mm. The green sheet thickness was set to 100 μm for Al_2O_3 and 80 μm or 40 μm for BS, creating a sandwich structure. The stacking conditions were set to 5 MPa, 60°C, and 1 minute using a manual stacker. The laminated structure was cut in the x-y direction using a blade cutter with a length of 30 mm. To remove excess organic materials within the laminate, it was heated at a rate of 3°C/min up to 600°C and held for 2 hours for binder burnout, followed by cooling. The sintering process involved heating the binder-burnt specimen on a zirconia substrate up to 1000°C at a rate of 2°C/min and holding it for 2 hours.

SI Note 2. Long-wave infrared region simulation

Figure S3 presents detailed demonstration of simulation-aided analysis for optical behavior underlying the low reflectance and high emissivity at the LWIR spectral range (8 ~ 13 μm). The wave response drastically changes within the LWIR wavelength region, since the refractive index of Al_2O_3 exhibits swift transition in the spectral realm. In the case of 8 μm wavelength, the real value of refractive index (n) maintains sufficiently high value while the extinction coefficient (κ) is nearly at zero. However, as the wavelength gets larger towards 13 μm at LWIR region, n value is suppressed to zero, while κ is gradually increased. In the case of low κ value, the incident electromagnetic wave successfully propagates into the deeper region of our model, due to the antireflective effect from its porous nature and without noticeable decadence. As the extinction coefficient increases, the amount of wave absorption at distributed Al_2O_3 particles accordingly increases, thus the wave is mostly dissipated at region near the air-structure interface.

Figure S3c and **Figure S3d** portraits calculated spatial reflected power at the air-structure interface of porous Al_2O_3 structure and dense Al_2O_3 film. Conventionally, general Al_2O_3 exhibits poor emissivity, and high reflection at the surface. However, owing to the porous structure, Al_2O_3 structure holds abundant void area between particles, thus mostly eludes reflection at structure surface and successfully absorb penetrated wave through particles surrounding pores. Hence, the porous structure exhibits low reflected power at the air-structure boundary. Measured spectral reflectance of the porous Al_2O_3 structure and dense Al_2O_3 film depicted in **Figure S3e** show significant correspondence to the preceding numerical analysis; spectral reflectance of the powder Al_2O_3 at wavelength region of 11 ~ 13 μm (where the κ drastically increases while the n swiftly decays) is way lower than that of the counterpart.

Additional field distributions in both model at wavelength of 8 μm, 11 μm, and 13 μm presented in **Figure S3f** and **Figure S3g** reinforces the analysis of wave behavior at different

configurations. Wave propagates towards deeper region in both structure at 8 μm. However, due to the antireflective effect occurring at porous structure, the field in the structure penetrates even deeper than the dense Al_2O_3 filled film, with less amount of dissipation. On the other hand, at wavelength of 13 μm, since the incident wave cannot infiltrate into the Al_2O_3 film structure, the wave is mostly scattered and absorbed at the surface, and exhibits high reflected power near the interface between air and the film. However, in the porous structure, the incident wave can slightly propagate into the void area between the Al_2O_3 particles, thus the wave is mostly scattered and absorbed at the pore area. Hence, the porous Al_2O_3 structure shows almost zero amount of reflected power at the air-structure interface.

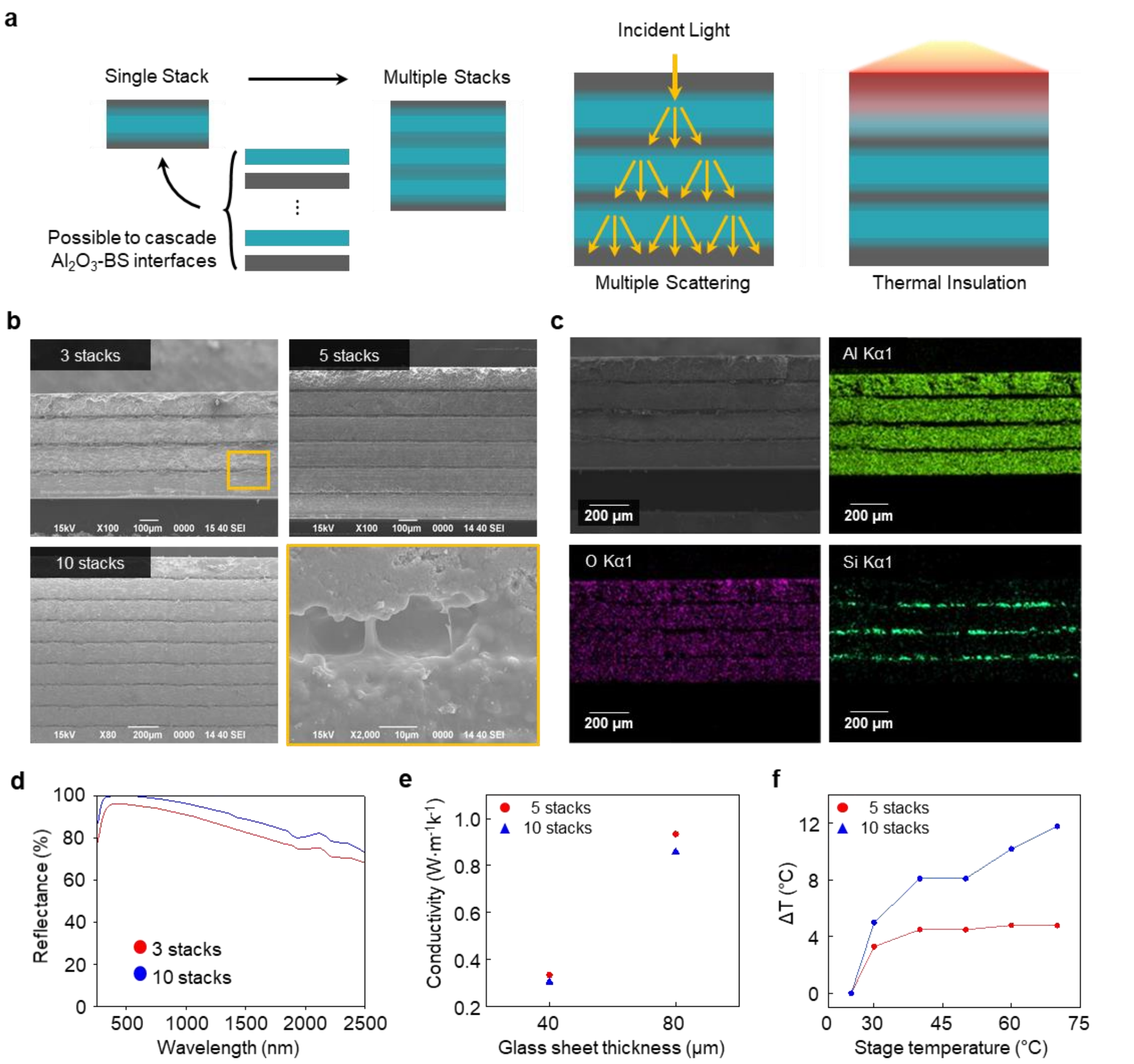

Figure S1. (a) Illustrations for the additional Al_2O_3-BS interfaces cascaded to the sandwiched structure. (b) SEM cross-sectional images of structures with different borosilicate stack numbers. The inset image outlined with red color shows the widened cross-sectional view of the single Al_2O_3-BS-Al_2O_3 interface. (c) The SEM cross-sectional image and the elemental map images of the panel highlighted for different elements are enlisted; aluminum (Al), oxide (O), and silicon (Si), respectively. Elemental maps reveal the spatial distribution of each element. Al and O elements are mostly distributed in alumina layer, while Si elements are mainly present in borosilicate layer. (d, e) The comparison of optical spectrum and thermal conductivity by

lamination thickness. (f) Temperature difference (ΔT) between the stage and ceramic composite with different layers plotted against stage temperature.

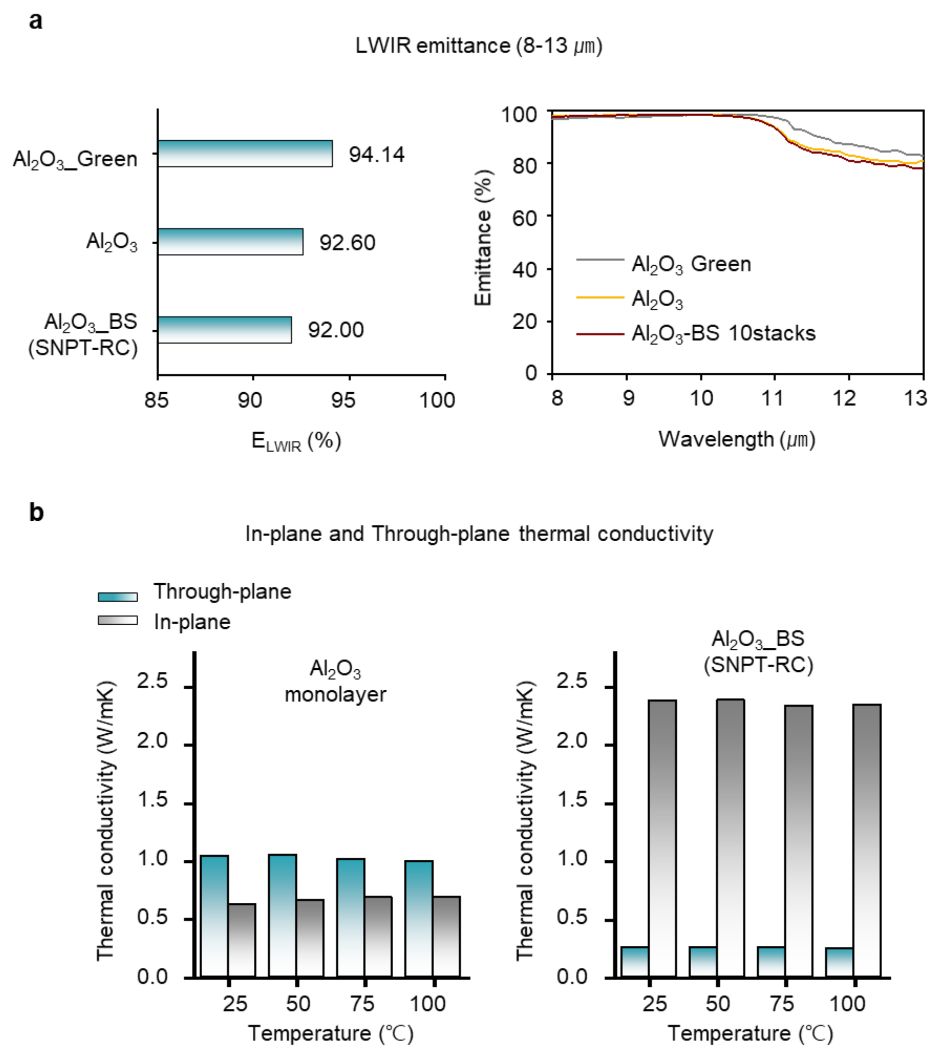

Figure S2. (a) LWIR emittance for Al_2O_3 green, Al_2O_3, and Al_2O_3-BS samples (left) and optical spectra in LWIR region (right). To enhance the thermal radiation into the universe, it is crucial for the surface to possess a significantly high LWIR emittance within the 8-13 µm range (E_{LWIR}).

$$E_{\mathrm{LWIR}} = \frac{\int_{8\mu\mathrm{m}}^{13\mu\mathrm{m}} I_{bb}(T,\lambda)\cdot \mathrm{E}(\mathrm{T},\lambda)\,\mathrm{d}\lambda}{\int_{8\mu\mathrm{m}}^{13\mu\mathrm{m}} I_{bb}(T,\lambda)d\lambda}$$

The spectral thermal emittance is represented by E(T, λ). Furthermore, $I_{bb}(T,\lambda)$ signifies the blackbody spectral radiance at temperature T, assumed to be 25˚C.

$$I_{bb}(T,\lambda) = \frac{2hc_0^2}{\lambda^5}\frac{1}{\exp\left(\frac{hc_0}{\lambda k_b T}\right) - 1}$$

Where, h is the Plank's constant, c_0 is the velocity of light, and λ is the wavelength, k_b is the Boltzmann's constant, T is the absolute temperature of blackbody. (b) In-plane and through-plane thermal conductivity for Al_2O_3 (left) and Al_2O_3-BS (right).

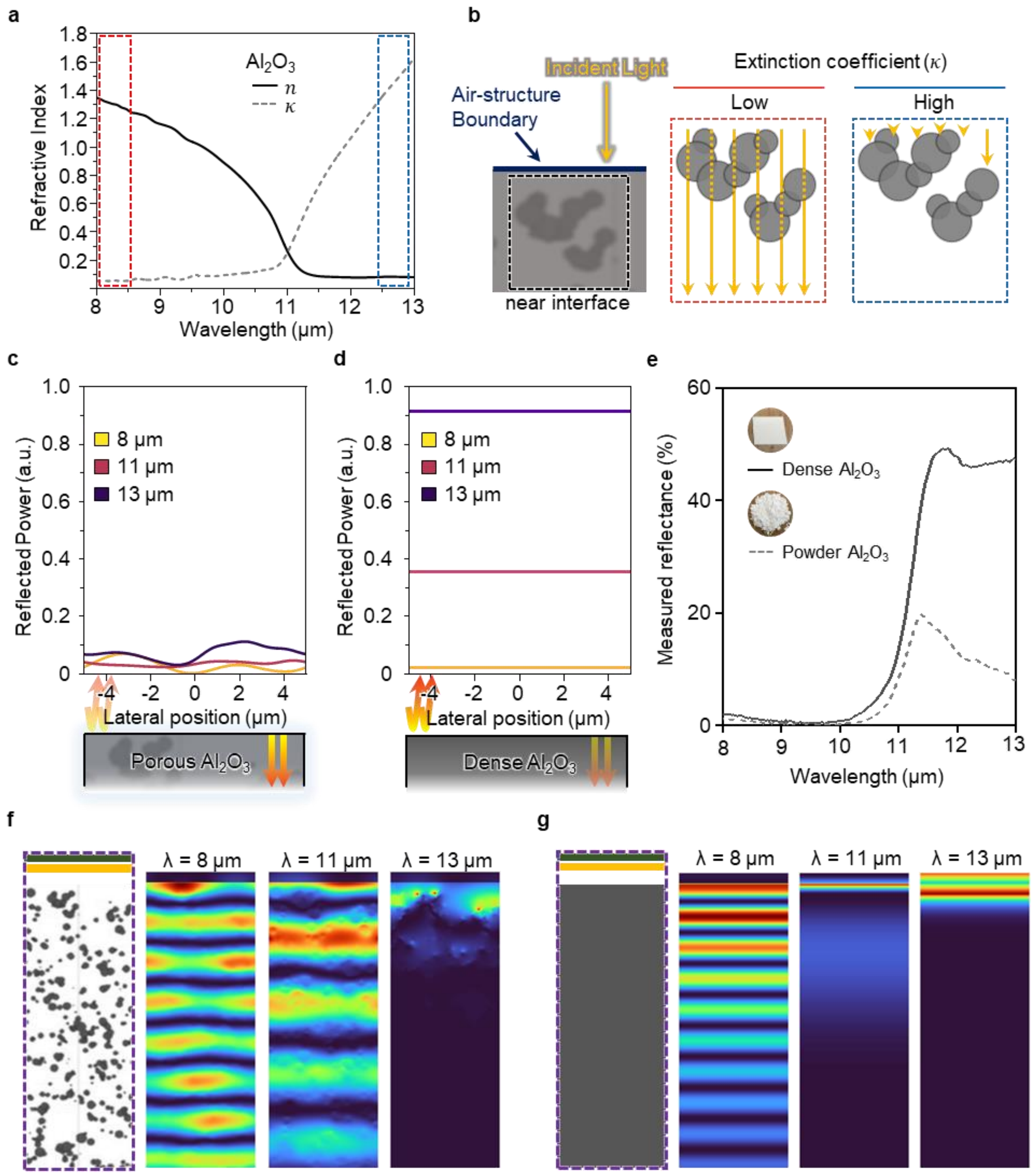

Figure S3. Simulation-aided analysis for optical behavior underlying the low reflectance and high emissivity at the LWIR spectral range (8 ~ 13 μm). (a) Refractive index of Al_2O_3 at LWIR spectral range. (b) Wave propagation behavior at area near the air-structure surface, with different κ value. (c, d) Calculated spatial reflected power at the air-structure interface. (e) FTIR spectrum for powdered Al_2O_3 and dense Al_2O_3 substrate. (f, g) Simulated field distribution for two structure model; porous Al_2O_3 structure and dense Al_2O_3 film. Detailed explanation is included in **SI note 2**.

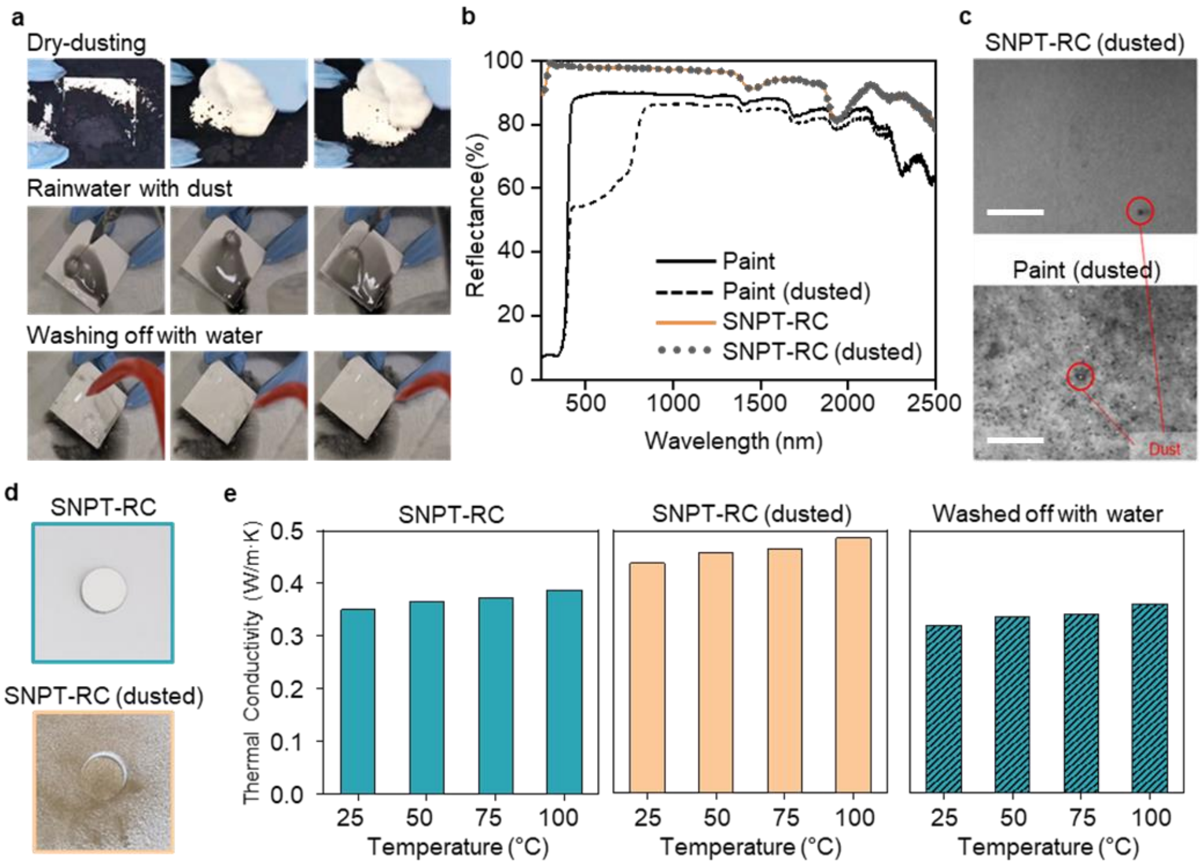

Figure S4. Examination on performance degradation of the SNPT-RC sample under pragmatic circumstances. (a) Photographs demonstrating dust scrubbing test. Each of rows demonstrate the sample being scrubbed with dust, defiled with rainwater mixed with dust, and washed off with water. (b) Optical measurement results of spectral reflectance for SNPT-RC and conventional paint sample, pure and dusted case for each of them. Unlike paint sample, the SNPT-RC doesn't suffer significant reflectance drop when dusted. (c) Optical microscopy of dusted SNPT-RC and conventional paint sample. The scale bar is 100 μm (d) Pictures of the pure SNPT-RC and the dusted counterpart. (e) Measured thermal conductivity with different temperatures, with changing the sample fouling configurations (*i,e.*, pure SNPT-RC sample, dusted SNPT-RC sample, and the sample washed off with water).

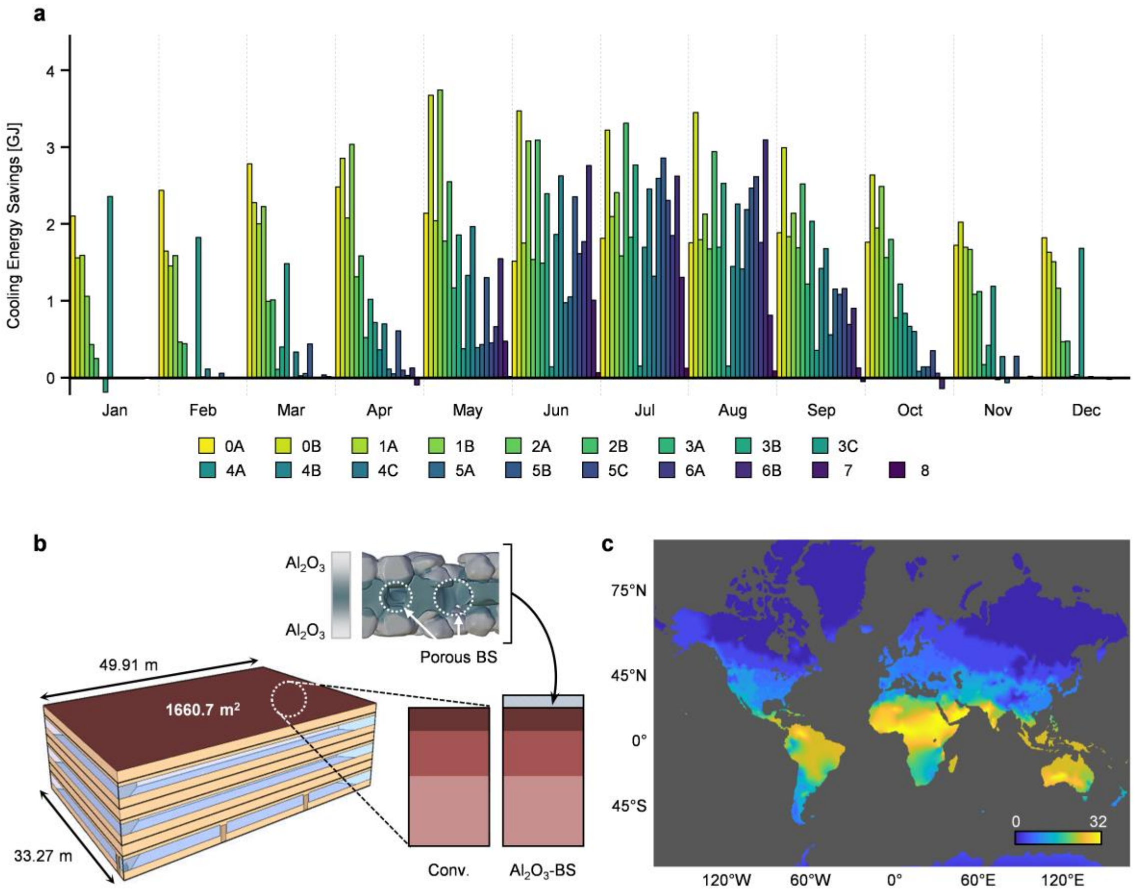

Figure S5. Global climatic cooling performance simulation. (a) Cooling energy savings of borosilicate Al_2O_3 outmost layered experimental buildings on monthly basis. For each month section, simulated cooling energy savings of 19 different climate zones (0A ~ 8) are plotted, sequentially. The detailed information about the conditions and specific representative locations of these climate zones are enlisted in **Table S1**. (b) Physical simulation structure of ASHRAE standard 90.1 (2019) commercial medium sized office. Cross-section of roof surface demonstrates the additional experimental Al_2O_3-BS layer stacked on the plain roof structure of conventional building. (c) Global heatmap of estimated annually saved cooling energy in GJ unit. The result shows intuitive figure of possible cooling energy efficient regions when our experimental setup is implemented.

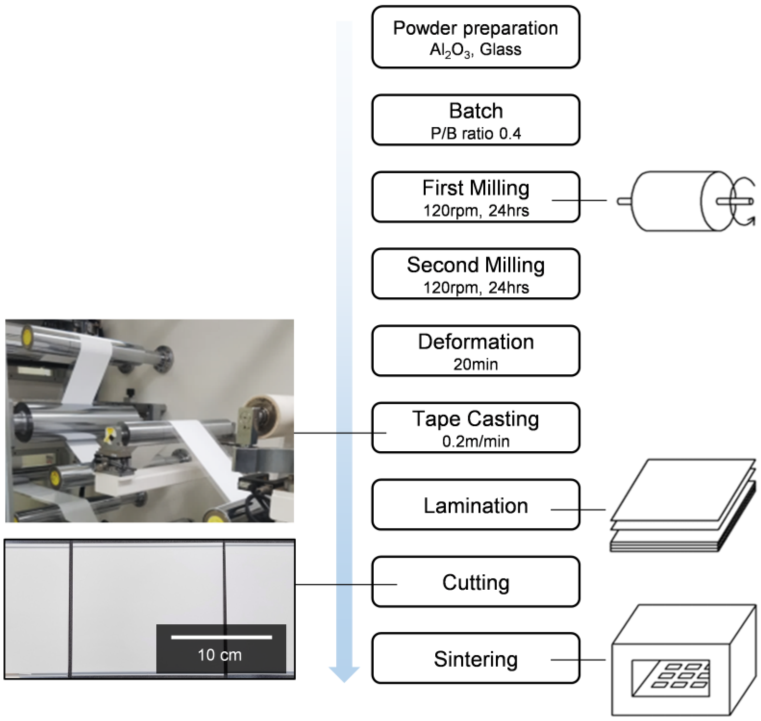

Figure S6. Fabrication process of SNPT radiative cooler. The entire fabrication processes of SNPT structure. In the batching process, the plasticizer-to-binder ratio is set as 0.4, and the batch is milled twice in total to produce a stable slip; for 24 hours at 120 rpm, each. The milling process is followed by a deformation process in order to obtain high-quality transparent alumina ceramic slip. Each of the layers in the SNPT structure is formed through the tape casting process and laminated in alumina-borosilicate-alumina order. The fabricated sample is cut into 10cm squared size, and annealed at high temperature, which allows the permeation of borosilicate particles into vacancies in neighbored alumina particles. Detailed process is demonstrated in **SI note 1**.

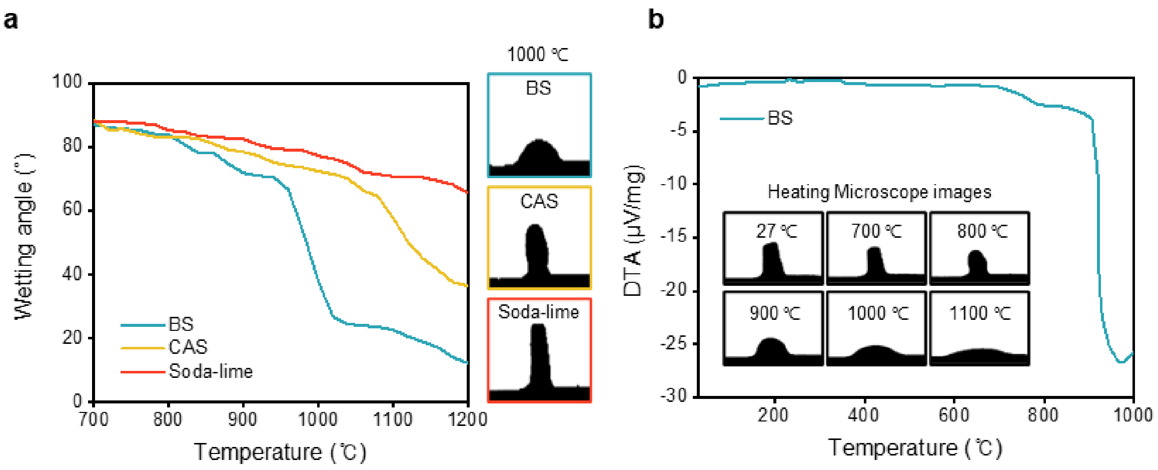

Figure S7. (a) Measured thermal characteristics of several glass materials for borosilicate (BS), calcium aluminosilicate (CAS), and soda-lime are displayed. The figure displays the quantified wetting angle of pillar-shaped structure constructed with three different glass materials over temperature varied from 700 °C to 1200 °C. Although the CAS and soda-lime structures tend to maintain its initial form even at high temperature, BS sample melts at comparatively lower temperature, around 900 ~ 1000 °C. Hence, for the perspective of a permeable feature, borosilicate shows superiority compared to other types of glass/ceramic material at the temperature when Al_2O_3 structure shows optimal optical condition, at the annealing temperature of 1000 °C. Additional differential thermal analysis (DTA) is conducted for detailed comprehension of the borosilicate's structural deformation through different temperature. At the temperature of 945 °C, DTA value suddenly drops, which implies that the pillar shaped sample of BS starts to melt and lose its solid state. The heating microscope images demonstrate the sample deformation with different thermal conditions. The borosilicate particle readily melts at the optimal annealing temperature of Al_2O_3 layer, which facilitates the successful infiltration of borosilicate particles into the spaces in Al_2O_3 porous layout.

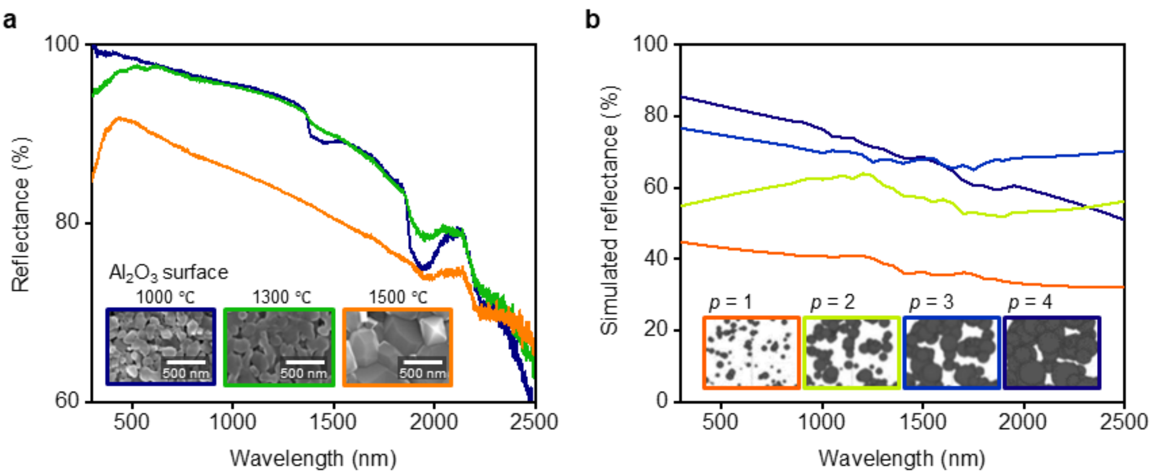

Figure S8. Optical characteristics of alumina and glass materials, and reflectance improvement by borosilicate permeation. (a) The transition of spectral reflectance for the Al_2O_3 structure with different annealing temperatures is presented. The annealing process of the Al_2O_3 monolith changes the shape of individual particles and negatively affect the optical properties. As demonstrated in three consecutive surface top view SEM images of porous Al_2O_3, particles melt and spread in size, bonding to other adjacent particles. Consequently, spectral reflectance tends to dwindle for higher annealing temperatures, due to the reinforced scattering of propagating waves at larger particles. (b) To understand the principle behind this phenomenon, further simulation is performed by calculating reflectance with the expansion of each alumina particle's radius; a factor p multiplied to the radius of the base particles is increased from 1 to 4, with an interval of 1. The spectra of reflectance are inclined to decrease as the size of particles gets larger. From this perspective, comparing the spectral reflectance of three different temperatures (i.e., 1000 °C, 1300 °C, and 1500 °C), the annealing temperature of 1000 °C is optimal for the alumina structure to exhibit efficient spectral reflectance.

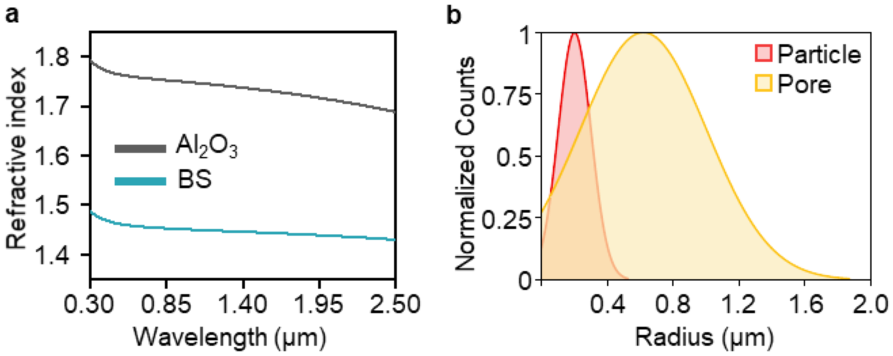

Figure S9. (a) The refractive indices of Al_2O_3 and BS, and (b) the radius distribution of alumina particles, and pores.

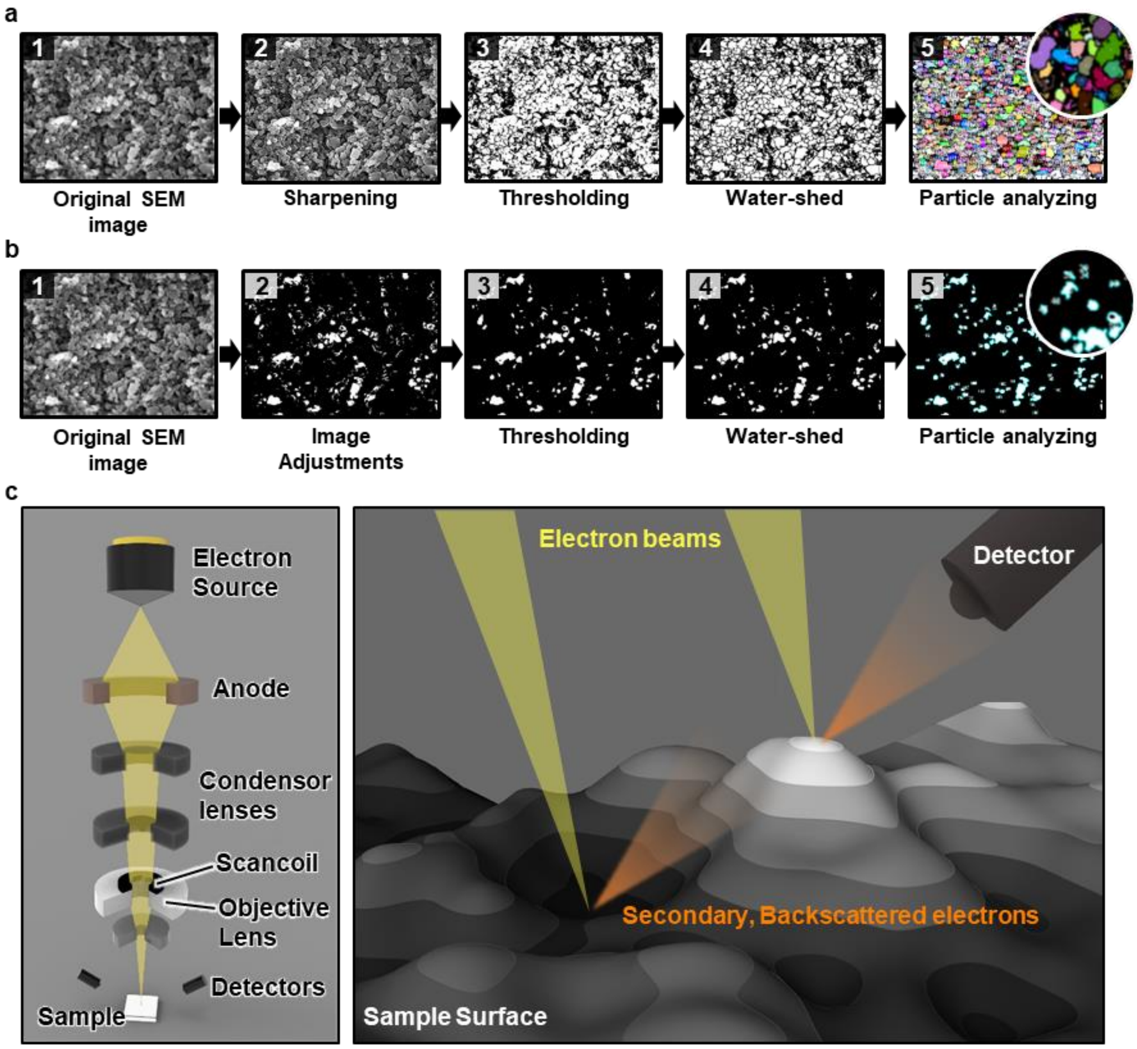

Figure S10. Detailed image processing procedure for modeling the Al_2O_3 layer. (a) The following steps are sequentially followed to determine the particle distribution on the surface onto which all the particles seen in the SEM image are projected: First, an original SEM image is sharpened to enhance the contrast of the entire image and to better differentiate different particles (Step 1). Then, the sharpened image is thresholded into binary and the particles are distinguished from the void background (Step 2). In the previous section, overlapped particles were presented as a single particle; thus, an additional water-shedding algorithm is implemented to separate discrete particles and resolve ambiguity (Step 4). By approximating the shapes of each captured particle as circles, the diameter of each can be defined from its perimeter (i.e., r = l/2π, where r and l represent the perimeter and radius of the particle, respectively) (Step 5). As a result of particle analysis, the data of particles that appeared on the original SEM image

can be extracted (i.e., particle counts, average, and standard deviation of the approximated particle radii). (b) Another SEM image analysis is conducted to exclude particles that are not located on the outermost plane. All procedures are identical to those executed in (a), except for Step 2, which sieves particles included in the outermost plane. Proper amounts of sharpening, brightness and contrast adjustments, and noise despeckling are applied to the original SEM image. Extracted white areas are assumed to be placed on an identical simulation plane, and subtle depth differences between separated white areas are neglected. (c) The SEM setup for inspecting the cross-section of the SNPT-RC structure and the mechanisms of analyzing surface contour are demonstrated in the depicted figures. Generally, particles with high proximity to the detector of SEM readily convey secondary and backscattered electrons compared to those located on the lowest area of surface dents. Thus, it is acceptable to regard white areas in the original SEM image as the highest points of the surface curvature. [42]

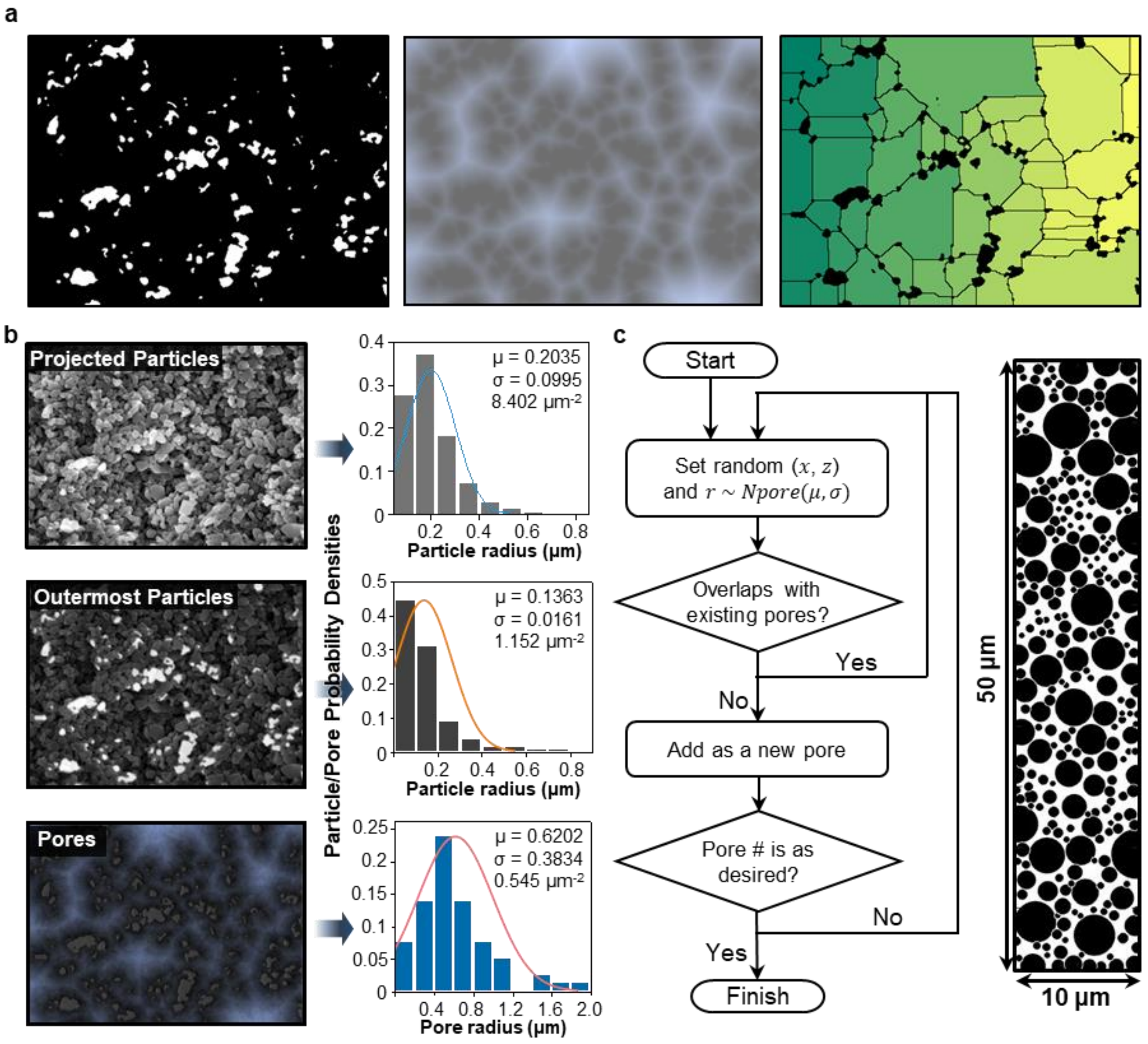

Figure S11. Analysis of pore distribution from SEM image and generation of pore filter. (a) The grayscale distance map shows the spatial remoteness of in-void points from the surrounding outermost particles. Areas with high contrast correspond to the farthest points from nearby particles, which could imply a high possibility of large pore placement in the area [43-45]. (b) Three plots present the statistics of projected particles, outermost particles, and pores, respectively. From these statistical values obtained from a partial area restricted to the SEM image region, we can estimate the structural features of the entire cross-section. (c) A logical diagram of the pore accumulation process and the resultant pore film is depicted. Every pore in the film must not overlap while maintaining the statistical characteristics we have previously obtained, namely the mean value and standard deviation of pore radius and the number of pores. For each iteration, the position of the additive pore is set randomly, and the radius is configured

with a Gaussian distribution, which agrees with the measured statistics from the image analyzing process. If the pore overlaps with other pores, the distance between the centers of the two circles is smaller than the sum of the two radii, and we skip assigning and start a new iteration if this is the case. The repetitive process terminates when the pore count reaches the desired number.

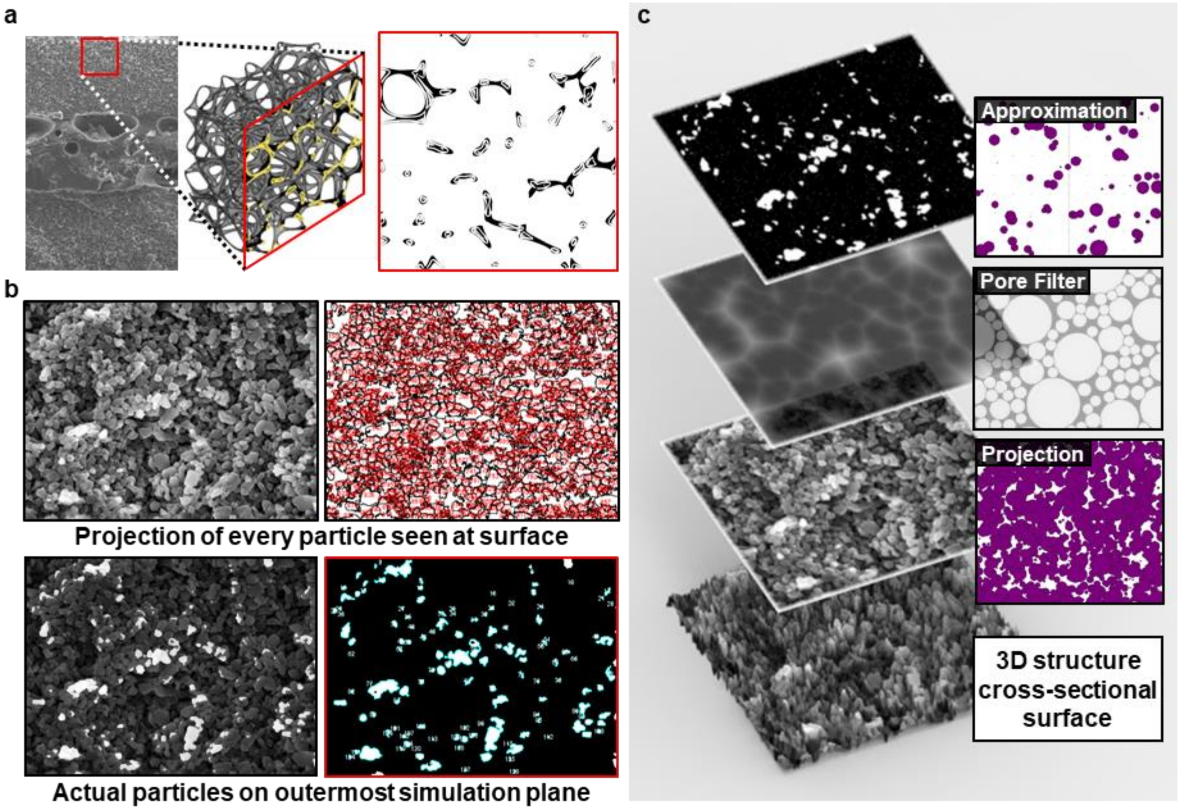

Figure S12. Comprehensive description of generating 2D simulation plane. (a) A region of the SNPT structure remote from borosilicate layer is mostly filled with Al_2O_3 porous structure, as demonstrated in **Figure 3**. From the cross-sectional image of a 3D porous structure model, one can roughly estimate how the Al_2O_3 particles are populated on a simulation plane. As depicted in rightmost figure, particles surround the sectional area of pore and are located in a loosely connected chain-like formation. (b) Two images are analyzed in particle-wise as demonstrated in **Figure S10**, which presents projection of particles seen at surface and actual particles located in outermost simulation plane. From the comparison between these two images, the voids in latter image are present mostly due to the porous structural environment. To be specific, one can almost conclude that pores have opted out the projection of particles not located in outermost simulation plane. (c) The approximated simulation plane can be generated by applying a pore filter on general projection plane. First, the projection plane is generated based on the extracted statistics of projected particles' radii. Particles are randomly positioned with compliance of previously determined number density, and sized according to Gaussian

distribution, which follows the average and standard deviation value of radii (**Figure S11b**, Projected Particles). Next, the pore filter is formed with randomly distributed non-overlapping circles, whose radii are also distributed to satisfy the pore statistics obtained from SEM image analysis (**Figure S11b**, Pores). Finally, the particles whose center coordinates are located inside the pore area of the filter (*i.e.* white areas) are opted out, and the resultant image is presented as the approximation plane image. Additional particle number management is conducted to fit with the resultant statistics of particles located in outermost plane of SEM image (**Figure S11b,** Outermost Particles), by randomly removing the excess amount of particles.

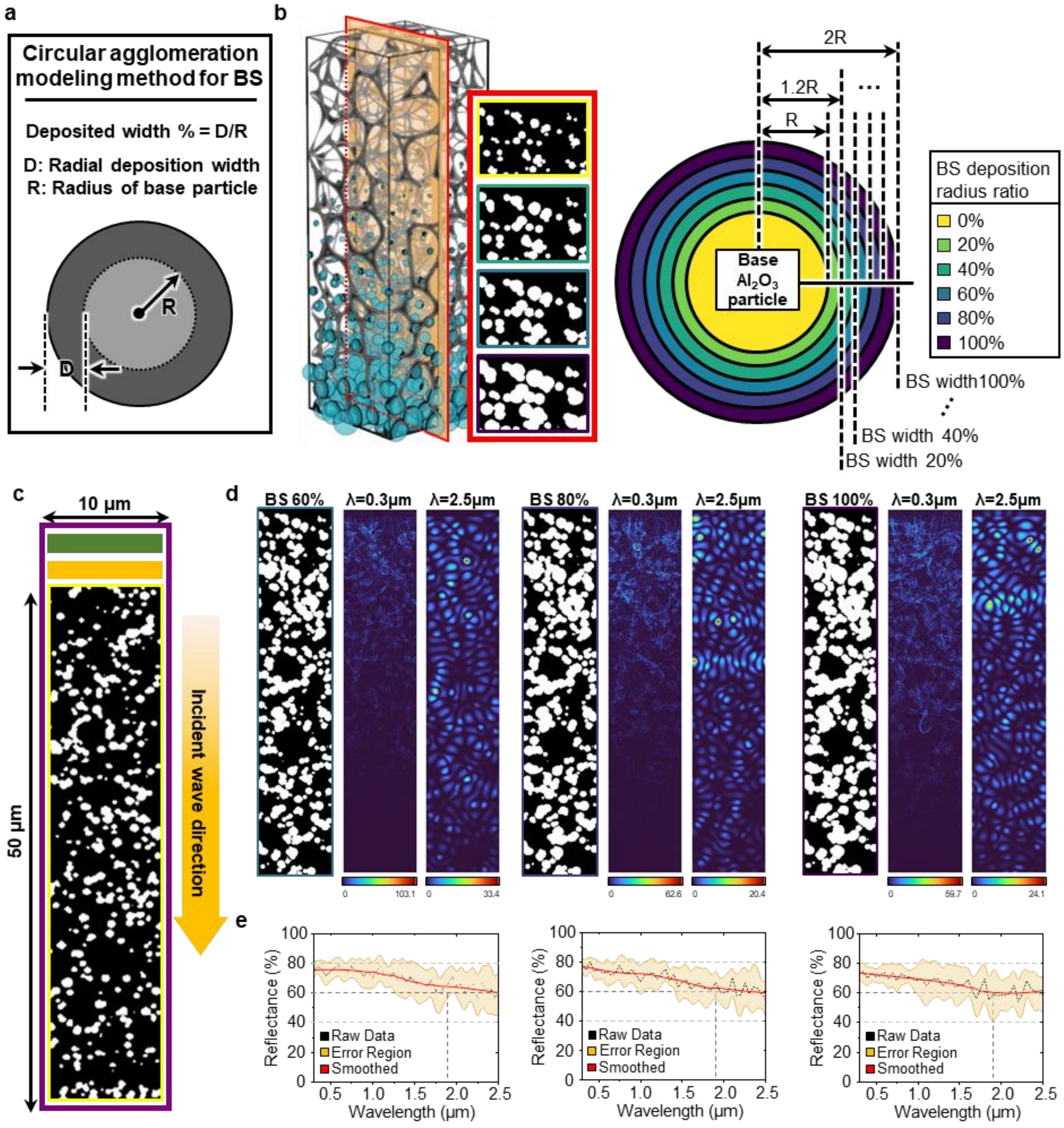

Figure S13. Supportive simulation results. (a) Borosilicate deposition is realized in the simulation by underlaying radially stretched borosilicate particle on the base Al_2O_3 particle, as demostrated in the figure. The Al_2O_3 particles are approxmiated to be circularly shaped. D is the partial deposition width of borosilicate in radial direction. R represents the radius of base Al_2O_3 particle. The percentage of deposited width is then presented by dividing the total deposition width with the radius of base Al_2O_3 particle. (b) The diagram demonstrates the planar simulation domain that includes the cross-sectional area of the SNPT-RC structure. As the region goes deeper to the alumina-borosilicate interface, the deposition rate gets higher. Four

indicative deposition rates (i.e. 0%, 40%, 60%, and 100%) are chosen to be simulated. The binary images of resultant 2-dimensionally modeled structure with regions of different depth are listed. White area and black background represents the particles and void spaces, respectively. For each of cases, additional layers with varied borosilicate deposition rate are shown at the right side of the binary images, and discerned with different colors. (c) The resultant simulation domain for the case of no borosilicate deposition is represented, with power monitor (green rectangle) and wave source (orange rectangle) on the top of entire simulation structure. The wave source and the monitor are located in order to properly measure the power of reflected wave. (d) Simulation structures are discretely generated for each aggregation rate of borosilicate, and electric field intensity simulation results are provided for two wavelength cases; 0.3 μm and 2.5 μm. (e) For each deposition rate, 10 different random structures are generated while satisfying the statistical condition for particle/pore radii and counts, obtained in the SEM image analyzing process. With these models, spectral reflectance is simulated separately, and the average values with errors are displayed spectrally. The resultant average spectral reflectance is then smoothed for better visuality. The simulated spectral reflectance for three cases excluded from **Figure 3** (*i.e.*, borosilicate radial deposition rate of 60%, 80%, and 100%) are depicted.

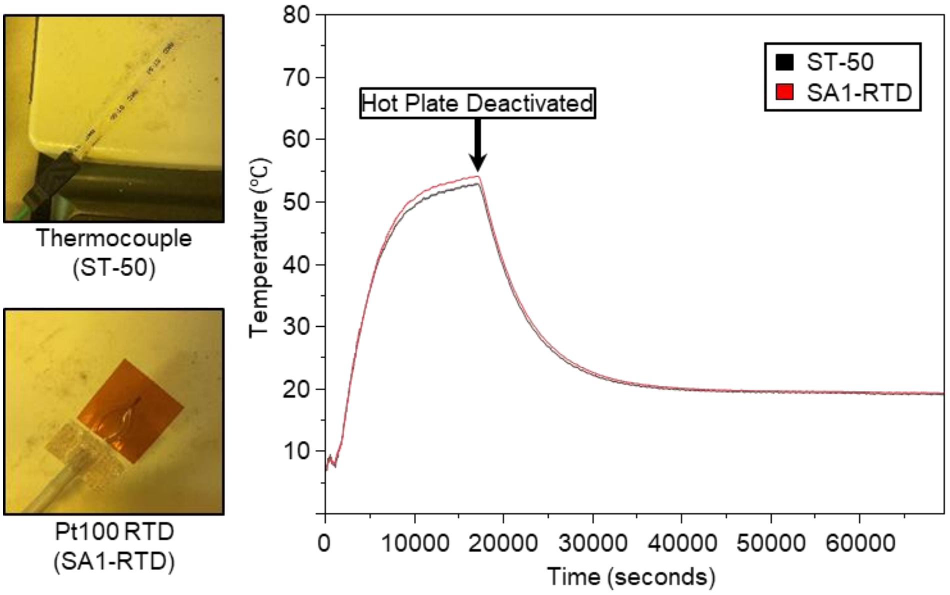

Figure S14. The thermocouple (ST-50, K-type) is verified to obtain the reliable sample temperature value. The Platinum-based resistance temperature detector (RTD) is used for the reference temperature value to figure out the accuracy of the thermocouple we used in the experiment. The reference RTD (SA1-RTD, Pt100 type) generally exhibits ~ 0.15 °C of error, thus sufficiently dependable to represent the temperature of a measured object. A cup of iced water exposed to heat source, and the temperature is measured using two thermal sensors; ST-50 thermocouple and SA1-RTD. Two sensors are attached on the inner wall of a beaker, and heat is inflicted to the iced water through a hot plate. The gauged value of ST-50 thermocouple compliantly followed that of Pt100 RTD. Although two values exhibit slight deviance of ~ 1.5°C nearly at point where hot plate is deactivated, they showed subtle difference in measured temperature value at steady state, within ~ 0.5°C. Considering that the experimental temperature circumstances in **Figure 4** mostly maintains to be stable, the temperature difference between two sensors are negligible. Thus, the thermocouple we utilized in the experiment (ST-50) is reliable to measure the temperature of the samples.

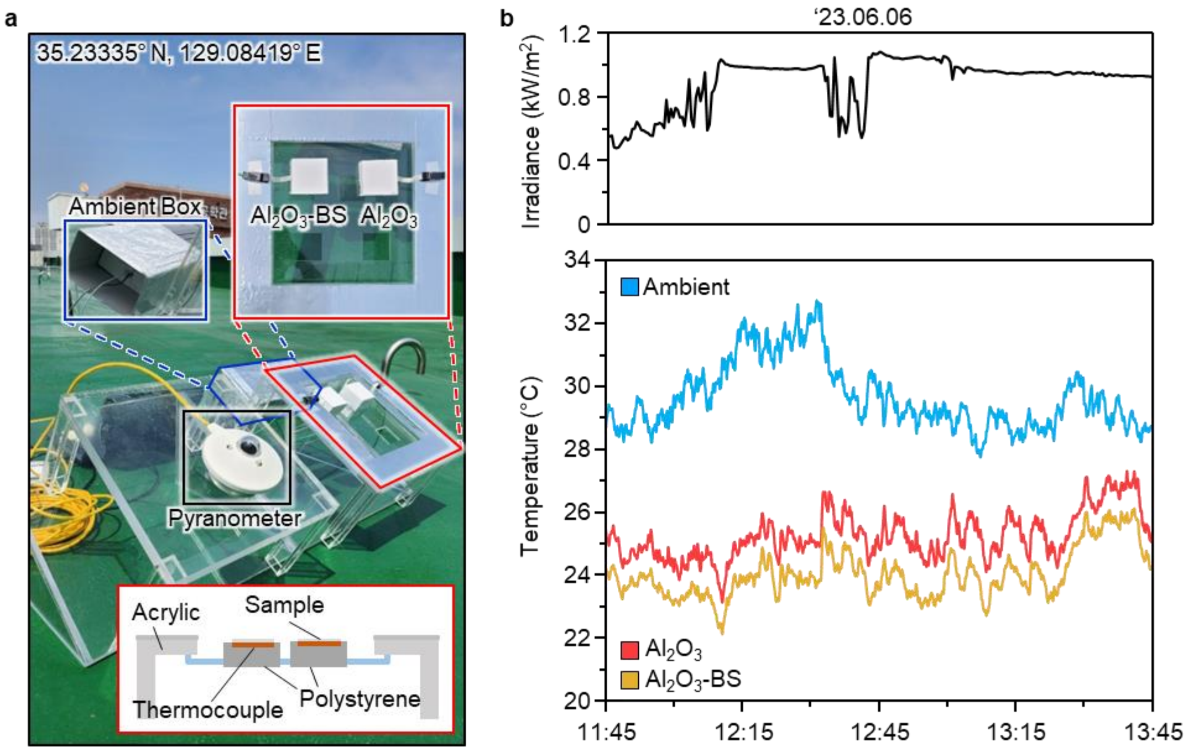

Figure S15. Outdoor measurement without PE film. To ensure the cooling performance of the radiative coolers under pragmatic application, temperatures of radiative coolers are examined under outdoor environment. (a) Experimental setup for solar irradiance and sample/ambient temperature measurement. The setup is configured with slope angle of 30°, directed towards the south direction. Pyranometer measures the solar irradiance under the configuration. Ambient box is designed to prevent the effect of radiant heat of the sun towards the atmospheric thermocouple probe. (b) Measured solar irradiance and sample/atmospheric temperature over elapsed time. The measurement took place at the rooftop of Pusan National University (35.23335° N, 129.08419° E). The temperatures and irradiance are measured from 11:30 AM to 1:00 PM during the daytime.

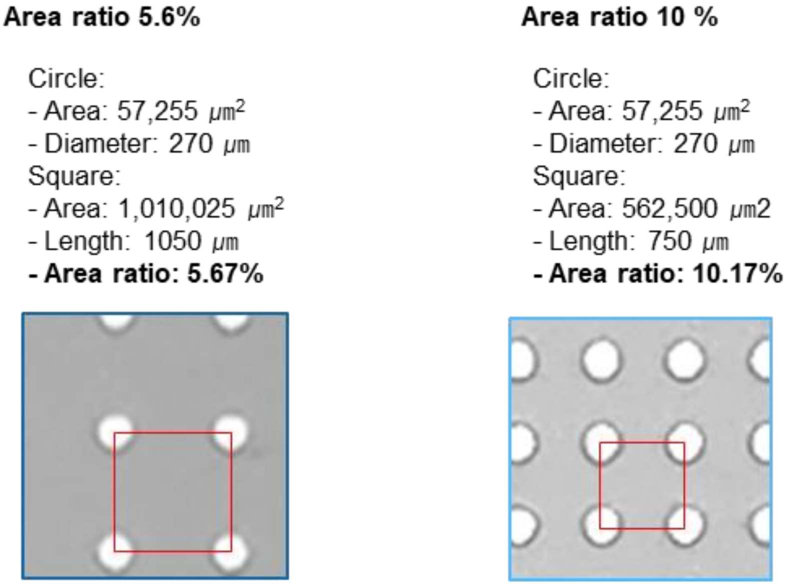

Figure S16. Through-hole areal ratio calculations with size configurations of areal ratio 5.6% and 10.17%.

Climate Zone Code	Climate Zone Name	Representative Location
0A	Extremely Hot Humid	Ho Chi Minh City/Tan Son Nhat Intl. AP, Vietnam
0B	Extremely Hot Dry	Dubai Intl. AP, United Arab Emirates
1A	Very Hot Humid	Kaohsiung Intl. AP, Taiwan
1B	Very Hot Dry	New Delhi/Safdarjun AP, India
2A	Hot Humid	Nanning, Guangxi, China
2B	Hot Dry	Cairo Intl. AP, Egypt
3A	Warm Humid	Tokyo, Japan
3B	Warm Dry	Amman Marka Intl. AP, Jordan
3C	Warm Marine	Cape Town Intl. AP, South Africa
4A	Mixed Humid	Seoul, South Korea
4B	Mixed Dry	Shijiazhuang, Hebei, China
4C	Mixed Marine	Seattle-Tacome Intl. AP, United States
5A	Cool Humid	Sapporo, Japan
5B	Cool Dry	Yinchuan, Ningxia, China
5C	Cool Marine	Van/Ferit Melen AP, Turkey
6A	Cold Humid	Montreal/Pierre Elliott Trudeau Intl. AP, Cananda
6B	Cold Dry	Hohhot, Nei Mongolia, China
7	Very Cold	Ekaterinburg, Sverdlovsk, Russia
8	Subarctic/Arctic	Magadan, Magadan, Russia

Table S1. Classification of different climates per ASHRAE standard, and corresponding representative locations. Several climate zones are classified in terms of temperature (extremely hot, hot, warm, mixed, cool, cold, very cold, subarctic) and humidity (humid, dry, marine). From climate zone code 0A (Extremely Hot Humid) to 8 (Subarctic/Arctic), there are total 19 different climate classes, and representative locations for each climate zone are enlisted on the rightmost column of the table.
